# On the intrinsic curvature of animal whiskers

**DOI:** 10.1371/journal.pone.0269210

**Published:** 2023-01-06

**Authors:** Yifu Luo, Mitra J. Z. Hartmann

**Affiliations:** 1 Department of Mechanical Engineering, Northwestern University, Evanston, Illinois, United States of America; 2 Department of Biomedical Engineering, Northwestern University, Evanston, Illinois, United States of America; Dartmouth College Geisel School of Medicine, UNITED STATES

## Abstract

Facial vibrissae (whiskers) are thin, tapered, flexible, hair-like structures that are an important source of tactile sensory information for many species of mammals. In contrast to insect antennae, whiskers have no sensors along their lengths. Instead, when a whisker touches an object, the resulting deformation is transmitted to mechanoreceptors in a follicle at the whisker base. Previous work has shown that the mechanical signals transmitted along the whisker will depend strongly on the whisker’s geometric parameters, specifically on its taper (how diameter varies with arc length) and on the way in which the whisker curves, often called “intrinsic curvature.” Although previous studies have largely agreed on how to define taper, multiple methods have been used to quantify intrinsic curvature. The present work compares and contrasts different mathematical approaches towards quantifying this important parameter. We begin by reviewing and clarifying the definition of “intrinsic curvature,” and then show results of fitting whisker shapes with several different functions, including polynomial, fractional exponent, elliptical, and Cesàro. Comparisons are performed across ten species of whiskered animals, ranging from rodents to pinnipeds. We conclude with a discussion of the advantages and disadvantages of using the various models for different modeling situations. The fractional exponent model offers an approach towards developing a species-specific parameter to characterize whisker shapes within a species. Constructing models of how the whisker curves is important for the creation of mechanical models of tactile sensory acquisition behaviors, for studies of comparative evolution, morphology, and anatomy, and for designing artificial systems that can begin to emulate the whisker-based tactile sensing of animals.

## Introduction

For most mammals, whiskers (vibrissae) are an important source of tactile sensory information, enabling exploratory behaviors that range from climbing [1] to locomotion [2], to hunting [3]. Whiskers emerge from richly innervated follicles in the cheek and are often arranged in a regular array of rows and columns [4]. Unlike insect antennae, whiskers have no sensory receptors along their length. Instead, when a whisker is deflected–either by direct physical contact or through fluid flow–the whisker transmits the mechanical deformation to its base [5–8]. The mechanical deformation is then transduced into electrical signals by different types of mechanoreceptors inside the follicle [9,10].

The geometry and material properties of a whisker will determine its mechanical response and thus the information conveyed to the animal’s nervous system. Both whisker bending and vibration are essential for whisker function [6,11–13], and these mechanical responses depend strongly on taper [6,14–18] and on the way that the whisker curves [5,19].

The taper of a whisker is well-defined in the literature: it describes how a whisker’s diameter changes along its arc length. As a first approximation, the diameter of a whisker tapers linearly from base to tip [15,20–23]. Higher resolution measurements have refined this approximation for the mouse, whose whiskers taper slightly more steeply in proximal regions (closer to the base) [24]. Similar high-resolution studies have not yet been performed for whiskers of other species.

In contrast to taper, which is well-defined, the way that a whisker curves is more challenging to quantify. In general, whiskers are planar–in other words, if a whisker is removed from an animal’s face and placed on a table, most of it will lie flat, though its most distal region will often bend slightly out of the plane [25–27]. The two-dimensional (2D, projected into the plane) shape of the whisker is termed its “intrinsic curvature” [5,24,26–30]. If the intrinsic curvature of a whisker were constant along its length, the whisker would assume the shape of a circle. However, intrinsic curvature is not constant, and thus various functions have been used to describe how it varies along the whisker’s length.

Approaches towards quantifying intrinsic curvature have varied considerably across studies. Previous researchers have approximated the intrinsic curvature of a rat whisker in a variety of ways: as a quadratic Bézier curve [31], as a piecewise polynomial function [32], as a parabolic function (y = Ax^2^+Bx+C) [25], as a parabolic function with only a quadratic term (y = Ax^2^) [26,27], and as a fifth-degree polynomial equation [14]. One recent study showed that the shape of rat whiskers can be mathematically transformed to fit intervals on the universal Euler spiral (κ = ŝ, where ŝ is the transformed arc length coordinate) [28], an approach that was later extended to include several other species [30].

The present work compares and contrasts different mathematical approaches towards describing whisker intrinsic curvature, and compares these approaches across ten different species: rat, mouse, gerbil, chinchilla, ground squirrel, rabbit, cat, harbor seal, sea lion, and pig. We discuss the advantages and disadvantages of each of the approaches. Constructing models of how the whisker curves is important for the creation of mechanical models of tactile sensory acquisition behaviors, for studies of comparative evolution, morphology, and anatomy, and for designing sensors and robots that can begin to emulate the whisker-based tactile sensing of animals.

## Materials and methods

### Ethics statement

All experiments involving animals and all tissue transfers were approved in advance by the Institutional Animal Care and Use Committee of Northwestern University. All work on tissue from marine mammals was performed with authorization from the National Oceanic and Atmospheric Administration’s (NOAA’s) National Marine Fisheries Service (NMFS) in accordance with Marine Mammal Protection Act regulations.

#### Data collection

A total of 1,703 whiskers from 10 species were used in this study (Table 1), including rats (*Rattus norvegicus*), mice (*Mus musculus*), gerbils (*Meriones unguiculatus*), chinchillas (*Chinchilla chinchilla*), ground squirrels (*Ictidomys tridecemlineatus*), rabbits (*Oryctolagus cuniculus*), cats (*Felis catus*), harbor seals (*Phoca vitulina*), sea lions (*Zalophus californianus*), and pigs (*Sus domesticus*).

**Table 1:** The number of whiskers and specimens used in this study.

Species	# of individuals	# of whiskers	Sex and age
Rat	4	225	All female, 5~36 months old
Mouse	7	323	All male, 6~8 weeks
Gerbil	2	172	2 female, 3 months old
Chinchilla	2	95	Unknown
Ground squirrel	3	116	Mixed sex, adult
Rabbit	2	126	Female, 10 months
Cat	6	228	Female, 11 months
Harbor seal	6	306	2 male, 4 female, all >1yr old
Sea lion	2	148	Female, subadult and 5+ years old
Pig	1	23	Female, age unknown

The whiskers from rats were the same as those used in previous studies [27,28]. Whiskers from mice, gerbils, ground squirrels, rabbits, and cats were collected immediately after the animals had been euthanized in unrelated experiments in other laboratories. Whiskers from harbor seals were obtained post-mortem in collaboration with Allied Whale at the College of the Atlantic (Bar Harbor, ME). Whiskers from sea lions were obtained post-mortem at the Marine Mammal Center (Sausalito, CA). One sea lion was classified as “subadult,” however, the lengths of its whiskers were indistinguishable from those of the adult specimen. All animals from all other species had fully grown whisker arrays.

For each animal, each whisker was identified by its row and column position (A, B, C, and so on from dorsal to ventral; 1, 2, 3 and so on from caudal to rostral), carefully plucked with fine forceps at its base, and then scanned in 2D on a flatbed scanner (Epson Perfection 4180 Photo) at 6,400 dpi resolution. Whisker shape was extracted as a 2D curve from the scanned image using custom-written software in MATLAB.

To reduce pixelation noise, each extracted whisker curve was smoothed using local regression (MATLAB “loess”) on a moving window of size 1,000 pixels (~4 mm). The whisker curve was then resampled into equally spaced nodes (0.25 mm) for analysis. In previous studies of rat whiskers [25–27], only the proximal 60–70% of the whisker was considered planar and used to quantify its shape. In contrast, the present work used the total length of each whisker for all species in all analyses. Although the three-dimensional (3D) whisker shape was effectively projected into the plane during scanning, the projected curvature closely approximated the 3D curvature for two reasons. First, none of the whiskers were observed to have any “twist” (i.e., they did not curl or kink). Second, an analysis of whisker planarity (S1 and S2 Figs) demonstrated that the out-of-plane curvature is typically quite small, and will have little to no effect on the assessment of intrinsic curvature.

#### Definitions: Whisker arc length, intrinsic curvature, and standard orientation

The “whisker arc length” is defined as the summation of the lengths of the segments formed by adjacent nodes along the trace. This metric is distinct from the straight-line base-to-tip length.

The “intrinsic curvature” of a whisker characterizes how curvature, κ, varies along the whisker arc length. In Cartesian coordinates κ is defined as

$$\kappa =\frac{\frac{d^{2}y}{dx^{2}}}{{\left(1+{\left(\frac{dy}{dx}\right)}^{2}\right)}^{1.5}}$$ (1)

Intrinsic curvature can be characterized either by fitting the whisker to a given curve model or by constructing models of κ itself. Although some representations of whisker shape are coordinate free, others rely on a choice about how to place the whisker in a Cartesian coordinate system. For example, evaluating κ using Eq (1) requires a choice about how to orient the whisker in Cartesian coordinates.

The “standard orientation” of a whisker in Cartesian coordinates is defined within the context of work that quantifies mechanical signals at the whisker base (e.g., [24,33–40]). In these studies, forces and moments are broken into axial and transverse components within a local (“whisker centered”) reference frame [37]. The whisker’s base is placed at the origin, its proximal region is aligned with the x-axis, and it is oriented concave up such that the majority of its area lies in the first quadrant.

To determine the standard orientation in practice, a previous study [27] performed an optimization to determine the fraction of the proximal region of the whisker that should be aligned with the x-axis. The optimization iteratively rotated the whisker such that between 1% and 30% of the proximal region of the whisker was aligned with the x-axis. The optimal percentage was determined by minimizing the mean squared error of a curve fit to y = Ax^2^. On average, error was minimized by aligning at a point 8% out along the whisker length, which was therefore used to align all rat whiskers with the x-axis.

**Fig 1:**
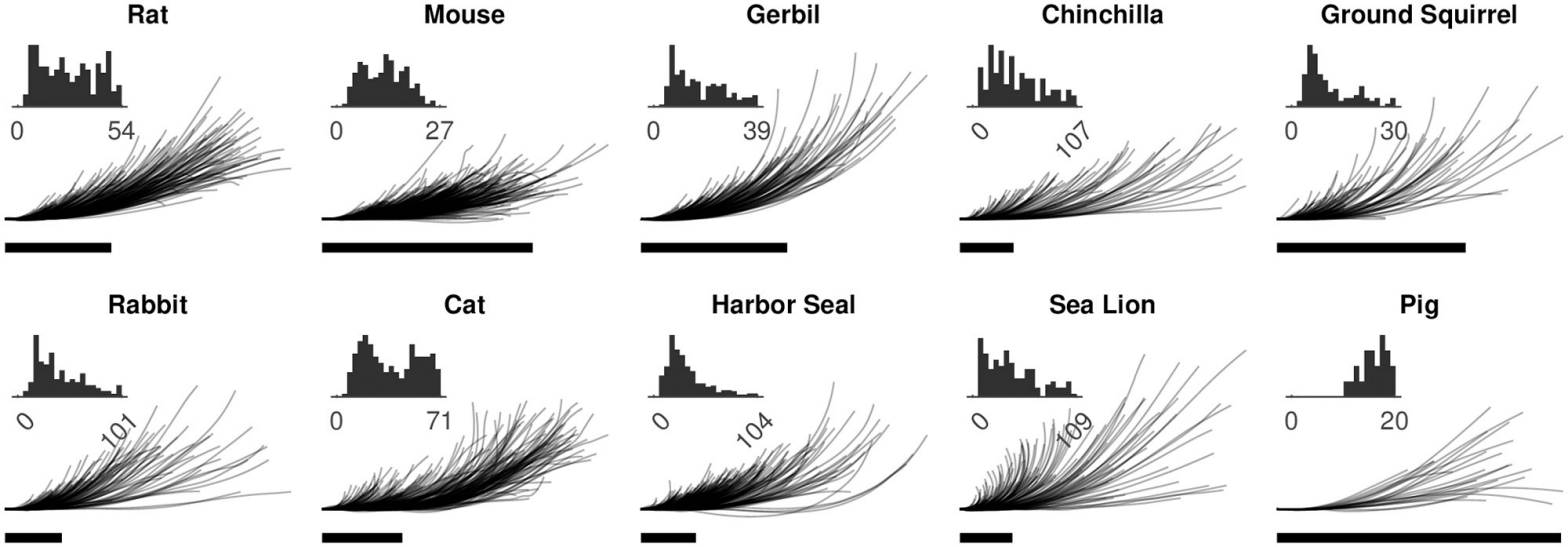
All whiskers are placed in standard orientation. In this figure, the proximal 15% of each whisker is aligned with the positive x-axis (horizontal). In the actual optimizations for different models, between 1~20% of whisker length was aligned with the x-axis. Scale bar: 20 mm. **Inset**: Histogram of whisker length (unit: mm). In each histogram the bin value adds up to the total number of whiskers shown in Table 1.

In the present study, standard orientation was optimized separately for each whisker within the curve-fitting optimization routine described in the next section. The percent of the proximal whisker aligned with the positive x-axis was constrained to lie between 1~20%. Fig 1 shows all whiskers in standard orientation, grouped by species. The inset histograms show statistics of whisker length.

#### Optimizing fits to different models of whisker curvature

Optimizations were run to fit the whiskers to each of the curvature models assessed in the present work. For each model, the equation for curvature was parametrically denoted as x(t; θ) and y(t; θ), where θ is a vector of unknown model coefficients. If the model relied on Cartesian coordinates, then the vector θ also included an angle α that determined the whisker’s orientation. The code is openly available in the online repository (https://doi.org/10.5281/zenodo.6967383).

As described in Materials and Methods, the whisker was sampled into equally-spaced nodes (0.25mm), denoted as (x_i_, y_i_), for i = 1, 2, …, n. In a preprocessing step, bounds were set on the range for α. The bounds were established by iteratively finding the angle required to align up to 20% of the proximal length of the whisker to the x-axis. The maximum and minimum values of these angles were used as bounds for the range of α. For each choice of model (polynomial, elliptical, etc…), the unknown set of parameters θ was then determined by solving two alternating optimization problems.

First, given θ in the current step, each point on the parametric curve (x(t_i_; θ), y(t_i_; θ)) closest to the observed point (x_i_, y_i_) was generated by minimizing

$$\underset{t}{\text{min}}\left[{\left(\hat{x}\left(t_{i};\theta \right)-x_{i}\right)}^{2}+{\left(\hat{y}\left(t_{i};\theta \right)-y_{i}\right)}^{2}\right]$$ (2)

where the parametric curve is rotated by α degrees. Note that orthogonal regression was used to account for observational error in both x and y dimensions.

Second, the optimal parameter set was found by minimizing the residual sum of squares (RSS):

$$\underset{\theta }{\text{min}}\overset{n}{\underset{i=1}{\sum }}\left[{\left(\hat{x}\left(t_{i};\theta \right)-x_{i}\right)}^{2}+{\left(\hat{y}\left(t_{i};\theta \right)-y_{i}\right)}^{2}\right]$$ (3)

The non-linear optimization of θ was performed using the built-in MATLAB (ver. R2020a) function “fmincon,” with the sequential quadratic programming algorithm specified. The optimization was terminated once the first-order optimality measure was met.

## Results

In the following sections, we compare and contrast multiple approaches towards quantifying the intrinsic curvature of whiskers from different species of mammals.

### Polynomial model

Polynomial equations were fit to each whisker from base to tip. This approach relies on orienting the whisker in a Cartesian coordinate system (see Materials and Methods). Because each whisker was placed in standard orientation before curve fitting, both the constant term a_0_ and the linear coefficient a_1_ are 0 by definition. Therefore, each whisker is fit with the following polynomial equation:

$$y=\overset{m}{\underset{i=2}{\sum }}a_{i}x^{i}$$ (4)

where m is the highest chosen order.

We noticed that when placed in the standard orientation, the tips of some whiskers curved so much that they went backwards along the x-axis. In these cases, the whiskers were virtually truncated at the point at which they began to curve backwards. This truncation is a disadvantage of the polynomial model, one that is avoided by the more complex models described in subsequent sections.

To prevent overfitting, we tested a variety of polynomial models of order increasing from 2. All whiskers were fit by the model y = a_2_x^2^, y = a_3_x^3^+a_2_x^2^, y = a_4_x^4^+a_3_x^3^+a_2_x^2^, and y = a_5_x^5^+a_4_x^4^+a_3_x^3^+a_2_x^2^, separately. For each model, the model coefficients and the RSS were obtained for each whisker. The rationale for choosing RSS as the metric for model selection is described in *Discussion*.

**Fig 2:**
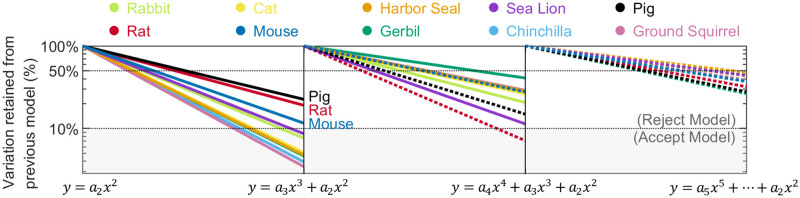
Decreases in the RSS from each lower order polynomial model to each higher order polynomial model are shown as a percentage of the lower order model (normalized to 100%). If a higher order model retains less than 10% of variation from the lower order model, it is accepted, otherwise it is rejected. Within each panel of the figure, species with solid lines are evaluated to determine if they would be overfit by the subsequent model. In the first panel, all lines are solid because all species are evaluated. The species pig, rat, and mouse are determined to be well fit by the model y = a_2_x^2^ and overfit by the model y = a_3_x^3^+a_2_x^2^. These three species are shown as dotted lines in the subsequent panel. The remaining seven species are shown as solid lines. These seven species are well fit by the model y = a_3_x^3^+a_2_x^2^ but overfit by y = a_4_x^4^+a_3_x^3^+a_2_x^2^ and therefore shown as dotted lines in the third panel.

Fig 2 shows, for all species, how the RSS for each whisker changes as the order of the polynomial fit increases. To avoid overfitting, a higher order polynomial model is rejected when it does not eliminate 90% or more of the variation of the previous model. Summarizing the results of Fig 2, a quadratic model y = a_2_x^2^ is sufficient for rats, mice, and pigs, but all other species require y = a_3_x^3^+a_2_x^2^. Any polynomial model with order higher than 3 is an overfitted model.

### Fractional exponential model

**Fig 3:**
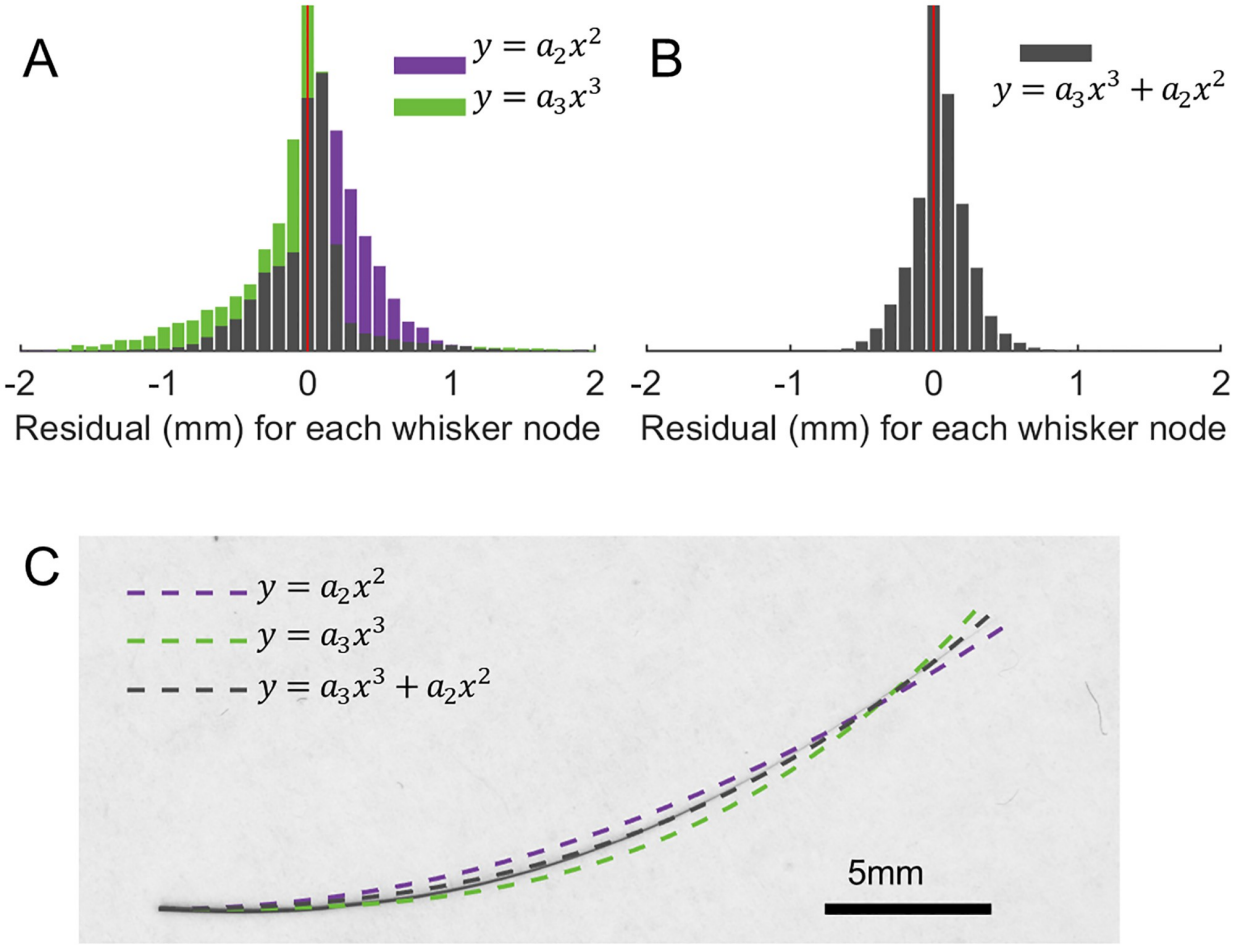
The quadratic and cubic terms in a polynomial model make different contributions to the fit. **(A)** The histograms of residuals for rat whiskers using the model y = a_2_x^2^ (purple) and y = a_3_x^3^ (green) are skewed to the opposite sides of 0 (red vertical line). **(B)** The histogram of residuals for rat whiskers using the model y = a_3_x^3^+a_2_x^2^ is shown; skewness is near zero. **(C)** Different curve fits are plotted for an exemplar scanned rat whisker. It can be observed that the quadratic and cubic models generate residuals on opposite sides of the whisker. The whisker shape lies between the two well-fit single-parameter curves, y = a_2_x^2^ and y = a_3_x^3^.

It is notable that the polynomial description of whisker curvature requires both quadratic and cubic terms for most species. Upon closer examination, it can be shown that these two terms contribute in different ways to whisker fitting. In Fig 3A and 3B, the residuals of the whisker fit are plotted as histograms for the models y = a_2_x^2^, y = a_3_x^3^, and y = a_3_x^3^+a_2_x^2^. The figure shows clearly that the residual histogram is biased in opposite directions for y = a_2_x^2^ and y = a_3_x^3^, and that the bias is less obvious for the polynomial model y = a_3_x^3^+a_2_x^2^, which combines the two terms.

Geometric intuition for these biased residuals can be obtained by examining an exemplar whisker (Fig 3C). This whisker is placed in standard orientation, and its shape falls between the two best-fit single-term polynomial curves, y = a_2_x^2^ and y = a_3_x^3^. A polynomial model combining the two terms provides a better fit–in other words, whisker shape is neither quadratic nor cubic.

Based on these results, we hypothesized that a model y = a_β_x^β^ with a fractional exponent β between 2 and 3 would better describe the general shape of a whisker than a polynomial model limited to integer exponent values. We hypothesized further, that the parameter β might provide a useful basis to quantify whiskers for each species, and perhaps differentiate between whiskers of different species. This analysis was performed through the following steps.

#### Step 1: Optimize fractional exponent values for *each* whisker

To find the optimal fractional exponent β value for each whisker as well as the model coefficient a_β_ and whisker orientation, we ran a nested optimization. An extra outside loop was added to the general optimization routine described in Materials and Methods. Recall that the vector θ includes a_β_ as well as the rotation angle α. In the outer optimization, given the optimized θ vector for a prior of β, the optimal β is obtained from:

$$\underset{\beta }{\text{min}}{\left(\overset{n}{\underset{i=1}{\sum }}r\left(t_{i};\theta ,\beta \right)\right)}^{2}$$ (5)

In the inner optimization (Eqs 2 and 3), the coefficient a_β_ was optimized based on the least RSS (∑r_i_^2^) given a fixed value for β. In the outer optimization (Eq 5), the optimal β value was chosen based on a slightly different parameter, namely, the overall minimal square of residual sum (∑r_i_)^2^. This metric accounts for bias by introducing product terms.

**Fig 4:**
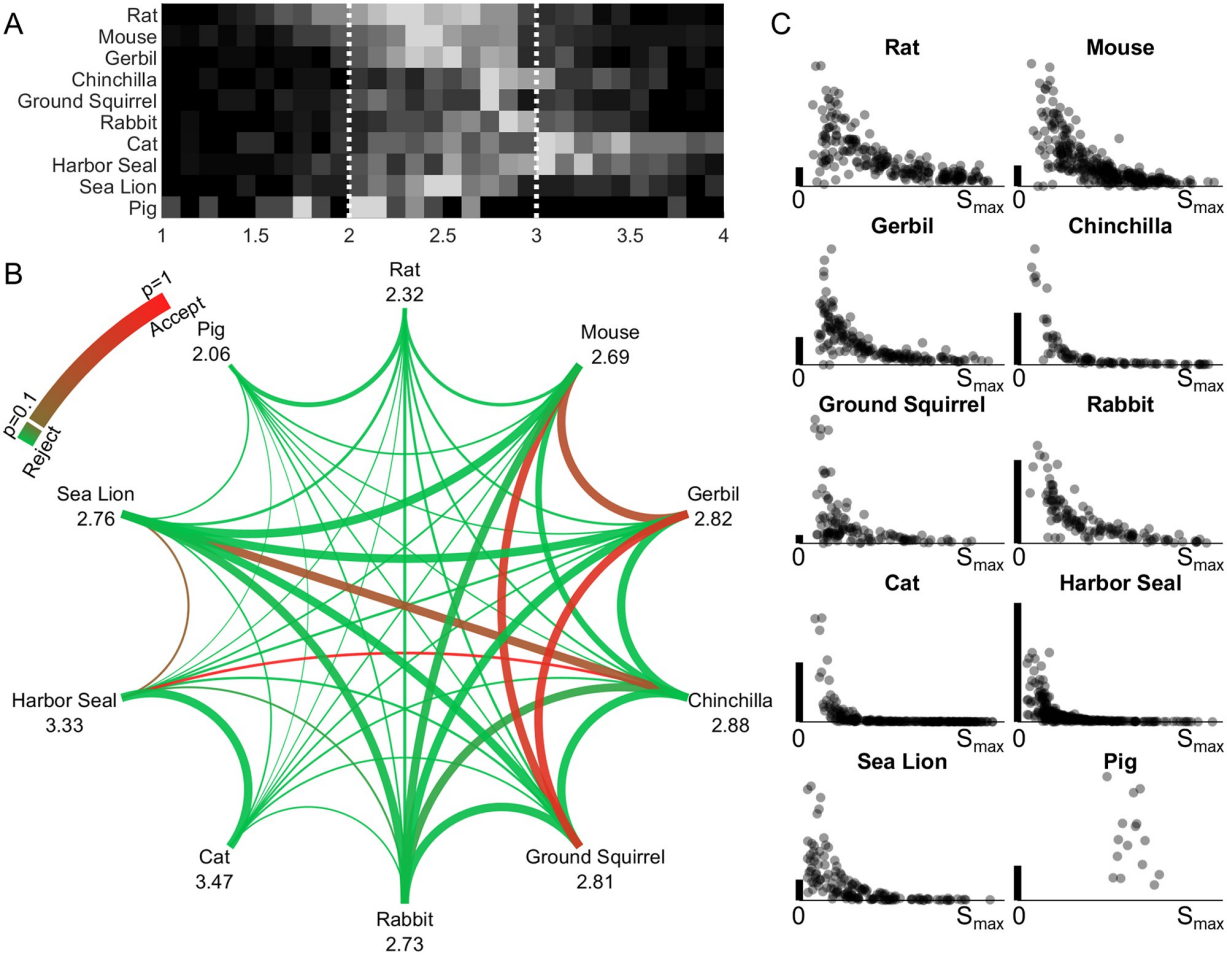
A fractional exponent model can be used to quantify whisker curvature. **(A)** A heatmap of exponent (β) values for whiskers of all species is shown. **(B)** This graph shows results of a cross-species comparison generated from the unpaired t-tests in step 2. The number under each species name is the generalized fractional exponent β value determined in step 3 (optimized across all whiskers for that species), and the thickness of each line connecting two species represents the difference in magnitude of the two β values, with thicker lines indicating more similar values. The color of the curve indicates the p-value of the student’s unpaired t-test. The green color rejects the null hypothesis, indicating that whiskers can be distinguished, while the red color accepts the null hypothesis, indicating that whiskers cannot be distinguished. **(C)** For each species, the coefficients a_β_ are plotted as a function of whisker arc length (S_max_). The values of a_β_ are highly variable for short whiskers compared to long whiskers. In all plots the vertical scale bar (heavy black vertical line) represents 0.005mm^1-β^ Each point is transparent gray so darker colors indicate a greater density of points.

In Fig 4A, the optimized fractional exponent β for each whisker is shown in a heatmap, with each row showing results for a single species. The fractional exponent values are mostly distributed between 2 and 3, confirming our hypothesis that the shape of most whiskers lies between quadratic and cubic curves and should be well approximated by the model y = a_β_x^β^ (2<β<3). However, Fig 4A also shows a non-negligible number of whiskers for each species with optimal values for β that are less than 2 or greater than 3. Whiskers with β values less than 2 are usually short and straight. Whiskers with β values greater than 3 have increases in curvature that are particularly large near the tip.

#### Step 2: Quantify significance of cross-species differences

In this analysis, whiskers with values of β outside the 95% confidence interval for that species were excluded to reduce variability (fewer than 5% were excluded for each species). The significance of cross species differences was then determined by conducting a student’s unpaired t-test (α = 0.05) between each pair of species. The null hypothesis is that the fractional exponent values of whiskers are from independent random samples from normal distributions with equal means and variances, that is, the whisker geometry is not separable between the two species.

Results of the hypothesis testing for all pairs are shown in Fig 4B. A total of 45 species pairs were tested. Using the value of the fractional exponent β as the only parameter, 39 species pairs are separable (p<0.1), of which 36 pairs are strongly separable (p<0.01). Six species pairs are not separable: mouse vs. gerbil (p = 0.47), mouse vs. ground squirrel (p = 0.71), gerbil vs. ground squirrel (p = 0.78), chinchilla vs. harbor seal (p = 0.97), chinchilla vs. sea lion (p = 0.38), harbor seal vs. sea lion (p = 0.23).

#### Step 3: Optimize the value of the fractional exponent β across each species, so that *all* whiskers from the same species share the same β value

This analysis was again performed using a nested optimization. It first minimized the overall RSS (∑r_i_^2^) of all whiskers for a given β value. Then the optimal β value with the least overall square of residual sum (∑r_i_)^2^ was selected.

In contrast to step 1, the fractional exponent model used in this step does not optimize the fit for each individual whisker, instead, it is optimized to describe the shape of all whiskers of a species. Species that were found to be indistinguishable in step 2 also have similar generalized fractional exponent values. These values are reported at the nodes of the graph of Fig 4B.

It is important to note that the fractional exponent β does not imply that it is possible to identify whether a particular whisker belongs to a particular species. Instead, β is intended as a general parameter to construct whisker shape for each species. Having generalized the β coefficient for each species, the variation of the a_β_ coefficient within a single species was then examined. Fig 4C shows the distribution of the a_β_ coefficient among whiskers of the same species. Short whiskers are likely to have larger and more variable values of a_β_, while longer whiskers have lower a_β_ values. These results are consistent with previous work [27] in which rat whiskers were fit to quadratic equations: a small change in the tip location for shorter whiskers will result in a large change in the a_β_ coefficient.

### Elliptical model

As indicated earlier, polynomial fits cannot accurately model whiskers whose tips go “backwards” (in the direction of the negative x-axis) when placed in standard orientation. We refer to these whiskers as “backwards whiskers.” Modeling the whisker as a segment of an ellipse can fit whiskers of this backwards type. We therefore considered the possibility that whisker curvature changes non-linearly from base to tip in a way that can be described by an elliptical model κ(x, y) = A^4^B^4^/(A^4^y^2^+B^4^x^2^)^3/2^. A point (x, y) is taken from a curve segment on an ellipse. The curve segment starts at the origin, initially moving along the positive x-axis, and going into the first quadrant. The equation for the ellipse is x^2^/A^2^+(y-B)^2^/B^2^ = 1.

This model has two parameters, A and B, corresponding to the lengths of major and minor axes, respectively. The model coefficients were fit for each whisker.

**Fig 5:**
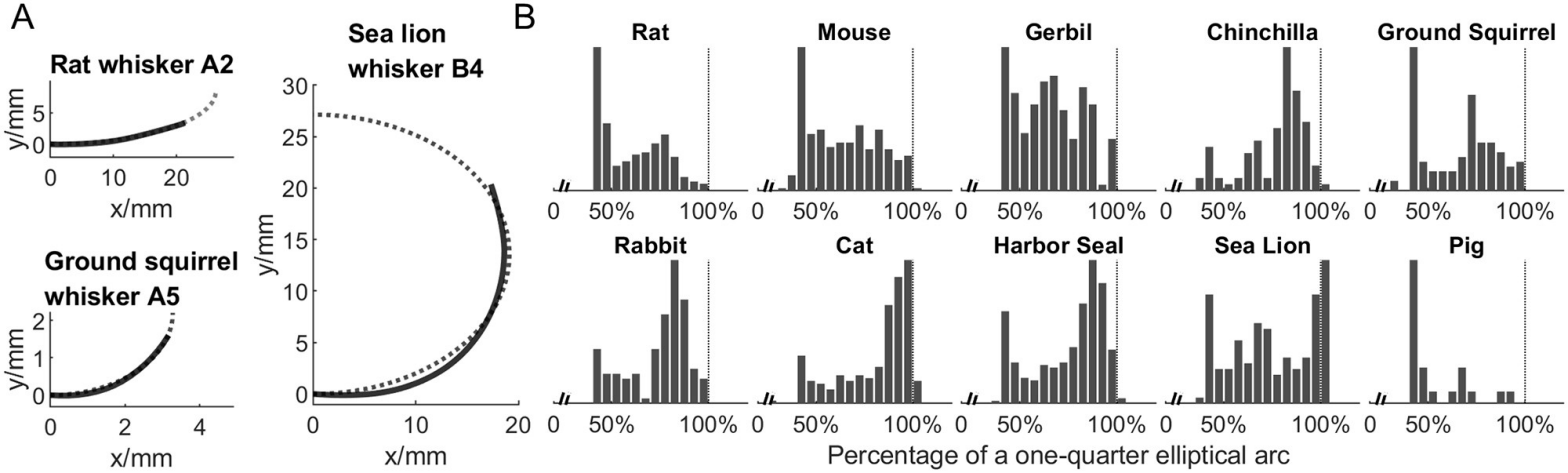
Results of fitting whiskers to an elliptical model are shown. **(A)** Three exemplar whiskers fit to ellipses are shown. Solid curve: Whisker. Dotted curve: Fit elliptical arc. **(B)** For each species, the histogram shows the number of whiskers that occupy a given percentage of a ¼ elliptical arc. Values greater than 100% (vertical dotted lines) indicate that the whisker occupied more than ¼ of the elliptical arc. The histogram bin value adds up to the total number of whiskers shown in Table 1.

Similar to previous analyses, an optimization process was run to determine the two coefficients, minimizing the RSS between each whisker and the fit elliptical arc. Fig 5A shows some exemplar whiskers and their best-fit ellipses. Most whiskers are fit within a single quarter of the elliptical arc, and some backwards whiskers can be accurately fit by extending the arc beyond one quarter. Fig 5B shows histograms of the percentage of the quarter-ellipse that is being used to fit the whiskers for each species. Backwards whiskers are observed as those occupying more than 100% of the quarter-ellipse (to the right of the dotted vertical line). It is notable that the minimum of all histogram bin values is ~40%, indicating that there are no cases in which a whisker was fit with a disproportionately large ellipse.

### Linear curvature model: Cesàro equation

Both polynomial and elliptical models rely on the choice of whisker orientation in a Cartesian coordinate system, but the Cesàro approach is coordinate free. Several previous studies have described rat whisker shape using a Cesàro model, in which curvature (κ) is assumed to change linearly with arc length (s) [26,28,30]. In other words, these studies have assumed that κ(s) = As+B, where A and B are coefficients that can be different for each whisker.

Although κ is typically defined in a Cartesian coordinate system (Eq 1), it is invariant under homogeneous transformation, rendering both A and B coefficients also coordinate-invariant. The sign of the coefficient A determines whether the curvature increases or decreases from a starting value B at the whisker base. We fit this model to the whiskers of all species in our dataset, optimizing the coefficients A and B by minimizing the summed Euclidian distance between each whisker and a curve of equal arc length generated by the equation κ(s) = As+B.

**Fig 6:**
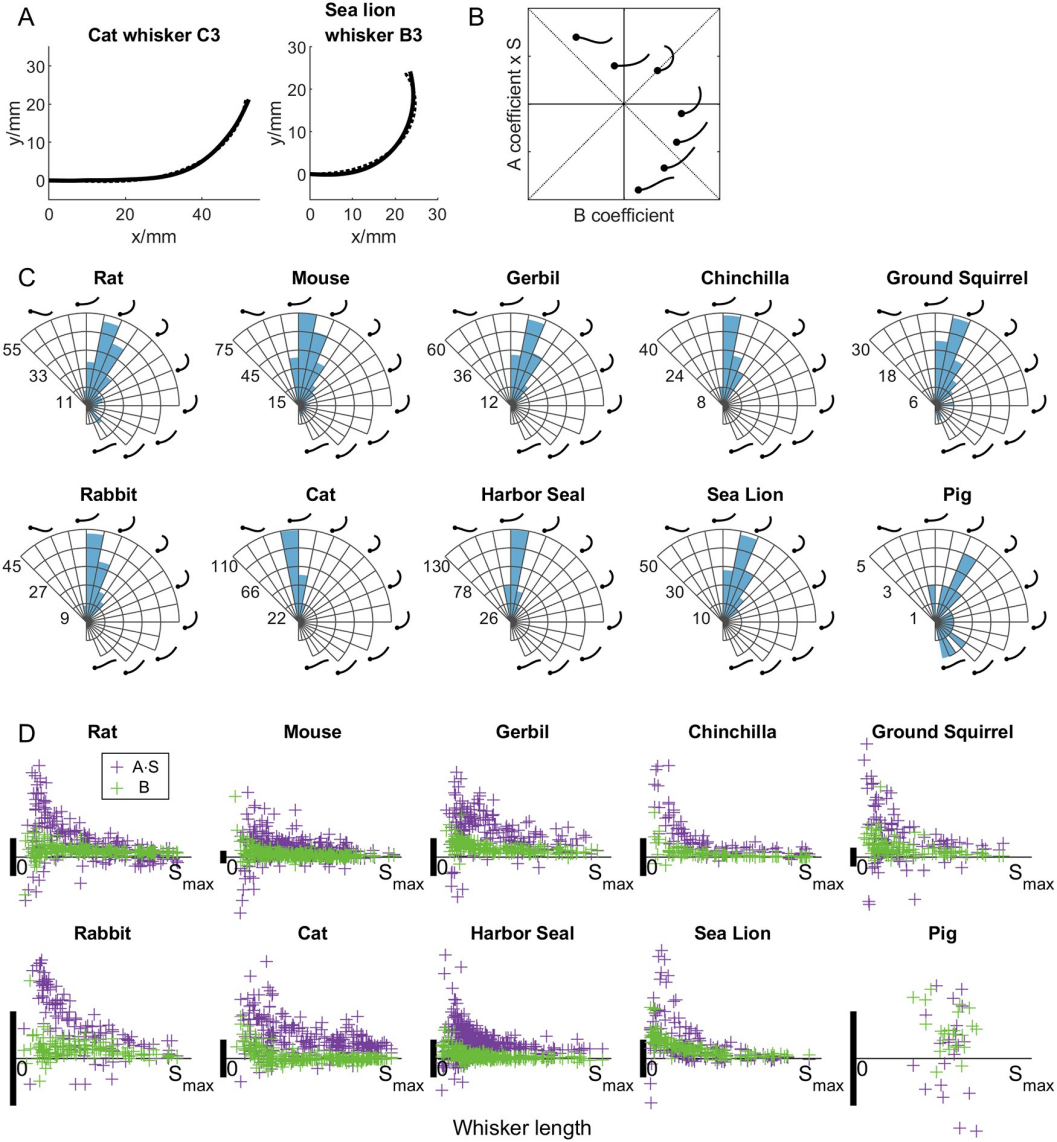
Whiskers can be fit to Cesàro curves. **(A)** Two exemplar whiskers fit to Cesàro equations are shown for cat and sea lion. Solid curve: Whisker. Dotted curve: Fit curve. **(B)** In the present dataset, whiskers could be classified into 9 different shapes, 7 of which are illustrated in the (B, A∙S) coefficient space (two are omitted in the first quadrant for visual clarity). The dots indicate the base of the schematized whiskers. **(C)** Polar histograms indicate the total number of whiskers in each region of the coefficient space for each species. The coordinates in each panel are the same as in (B). The radial axis indicates the number of whiskers. **(D)** The two coefficients B and A∙S show large variance for short whiskers compared to long whiskers. In all plots the vertical scale bar (heavy black vertical line) represents 0.05mm.

Whisker shapes are generally well represented by curves with a linear rate of change of curvature. Fig 6A shows two exemplar whiskers, from a cat and a sea lion. Linearly changing curvature can capture a wide variety of whisker shapes. With only two parameters, curve fitting works for whiskers that are approximately quadratic or cubic, as well as for whiskers that are “flat” at the beginning and curved up towards the tip (typical of many cat whiskers), and also for whiskers in which the tip goes backwards (typical of many sea lion whiskers).

Given the Cesàro equation κ(s) = As+B, the general shape of a curve can be categorized into 16 types. In our dataset, we found that the shape of a whisker (from any species) was limited to nine of these 16. The relative magnitudes of the two coefficients determine the shape of the curve; seven distinct examples are schematized on the plot of coefficient space in Fig 6B.

After optimizing fits for all whiskers across all species, the majority (74.3%) of whiskers were found to fall into the first quadrant of the coefficient space, as shown in Fig 6C. For these whiskers, the curvature starts with a positive value at the whisker base (B>0) and increases towards the tip (A>0). A smaller number of whiskers (14.6%) were found to lie in the second quadrant (B<0, A>0), and fewer (11.0%) in the fourth quadrant (B>0, A<0), in which curvature gradually decreases from the base. In our dataset, only two out of 1762 whiskers were found to lie in the third quadrant. Across whiskers of all species, 80.3% have positive curvature all the way from whisker base to the tip.

We next examined whether the distribution of A and B coefficients were different for long and short whiskers. Fig 6D shows the coefficients plotted against whisker arc length. Across all species except the pig, shorter whiskers tend to have higher variability in both coefficients than longer whiskers. The large variation shown for short whiskers is also consistent with that shown for the fractional exponent model and with results from previous studies [27].

### Conforming whiskers onto a universal Euler spiral

Curves described by Cesàro equations (κ = As+B) are called “Euler spirals.” The particular curve defined by κ(s) = s (A = 1, B = 0) is referred to as the “universal Euler spiral,” the parametric equations of which are given by Fresnel integrals, namely $x\left(t\right)=\int_{0}^{t}\text{cos}\left(\tau^{2}\right)d\tau$ and $y\left(t\right)=\int_{0}^{\tau }\text{sin}\left(\tau^{2}\right)d\tau$. A recent study [28] has shown that the shape of rat whiskers can be mathematically transformed onto arc segments of different lengths at different locations on the universal Euler spiral.

**Fig 7:**
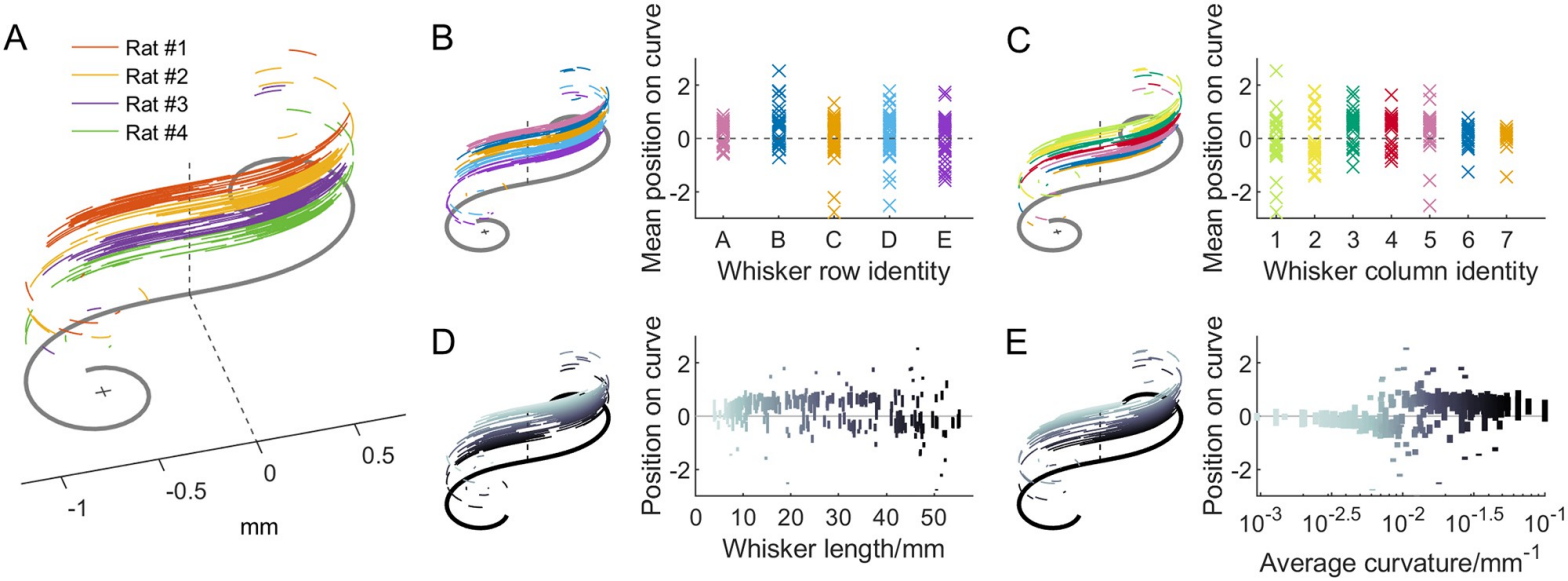
Whiskers are conformed onto the universal Euler spiral. **(A)** The heavy gray line indicates a universal Euler spiral. A total of 225 whiskers from four rats are conformed onto this spiral and vertically offset so that they can be visualized clearly. Color indicates whiskers from four different rats. **(B)** *Left*: Whiskers are mapped onto the Euler spiral and sorted (vertically offset) by their row identity. The conformed whiskers overlap almost completely (distinguishable only by the offset), indicating that the mapping shows no correlation to whisker row identity. *Right*: The mean position of the whisker on the Euler spiral is plotted as a function of row. **(C)** Identical to B, but for column identity instead of row identity. Again, the mapping shows no correlation to whisker column identity. **(D)** *Left*: Whiskers are sorted (vertically offset) by their arclength and mapped onto the Euler spiral. The grayscale matches the color of the arc length indicated in the right panel. *Right*: Each vertical line shows the span of the whisker conformed on the universal Euler spiral. No correlation is observed between arc length and either the mean location or the span of the whisker on the Euler spiral. **(E)** Identical to (D), but for average whisker curvature instead of arc length.

Fig 7A replicates the results for rat whiskers shown in previous work [28]. The mean locations of the conformed whiskers on the Euler spiral do not show strong correlations with either row (Pearson correlation test, ρ = -0.109) or column (ρ = 0.102), or with whisker arc length (ρ = -0.141), or with the curvature averaged over the arc length ( = A;S/2+B, where S is the total length of the whisker. ρ = 0.152) (Fig 7B-7E). All of these quantities are mapped in a non-systematic way onto the universal Euler spiral. In other words, transforming the whiskers to lie on the Euler spiral loses information about whisker row and column identity, whisker arc length, and average curvature over the arc length.

Thus while it is true that each whisker can be mathematically transformed so as to lie on a universal Euler spiral, that fact would be true for any curve that follows κ = As+B, and the value of doing so is not immediately clear.

### Comparing modeling approaches

**Fig 8:**
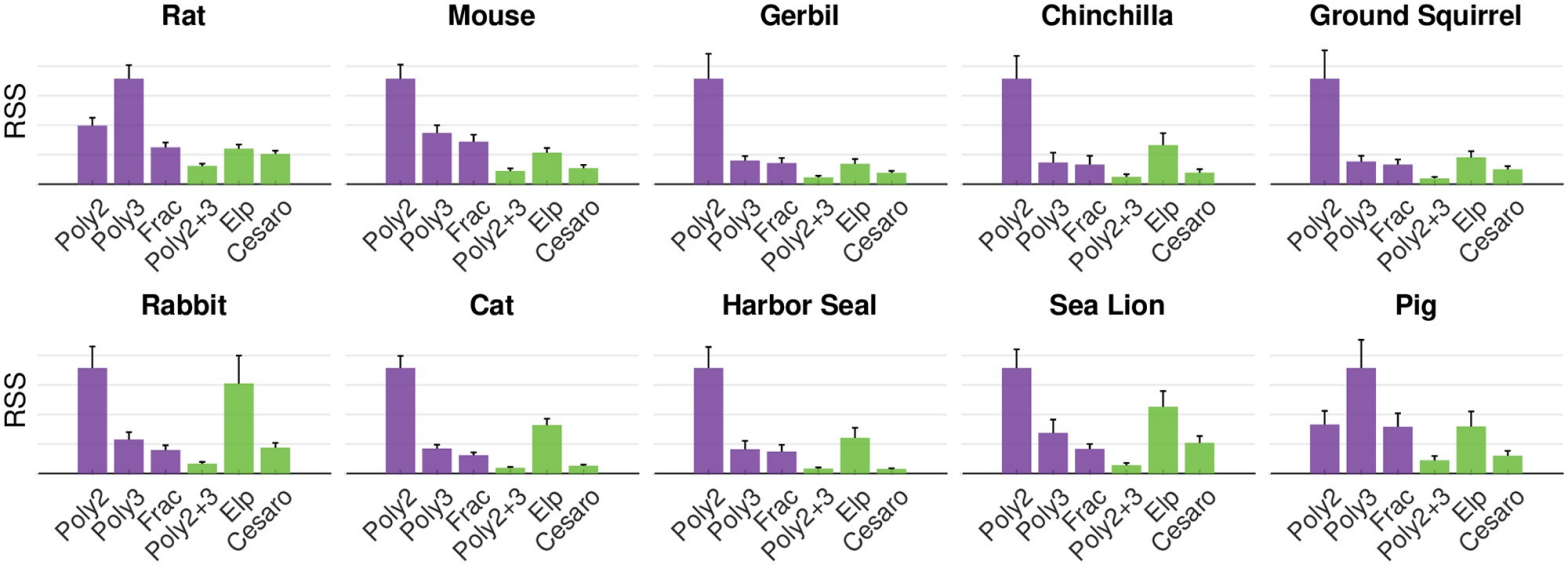
RSS is compared for all models for all species. In each subplot, the y-axis is normalized to the maximum value. All plots show adjusted RSS to account for differences in the number of parameters (either one or two). Poly2: y = a_2_x^2^. Poly3: y = a_3_x^3^. Poly2+3: y = a_3_x^3^+a_2_x^2^. Frac: Fractional exponent model. Elp: Elliptical model.

Fig 8 evaluates the different model types for all species using the RSS as a metric after removing outliers for each species (fewer than 3% on average).

Unsurprisingly, models with two parameters tend to have lower values of the RSS than single parameter models. The two-parameter polynomial model and the Cesàro fit always have a lower RSS than the single-parameter models y = a_2_x^2^ and y = a_3_x^3^. However, the elliptical model (two parameters) does not always have a lower RSS than the single-parameter polynomial models, and it does not appear to be a good choice for any species.

Within two-parameter models, the polynomial model has lower RSS values than the elliptical model and the Cesàro model for all species except the harbor seal. Overall, the best single parameter model is the fractional exponent, while the best two-parameter model is either polynomial or Cesàro.

### The method used to quantify a whisker’s intrinsic curvature affects the analysis of whisker mechanics

Any analysis of the mechanics of whisker-based sensing behaviors will be influenced by the choice of model for whisker curvature. As a simple example, we show how different models for whisker curvature affect an analysis of how an animal might determine the radial distance at which the whisker makes contact with an object.

**Fig 9:**
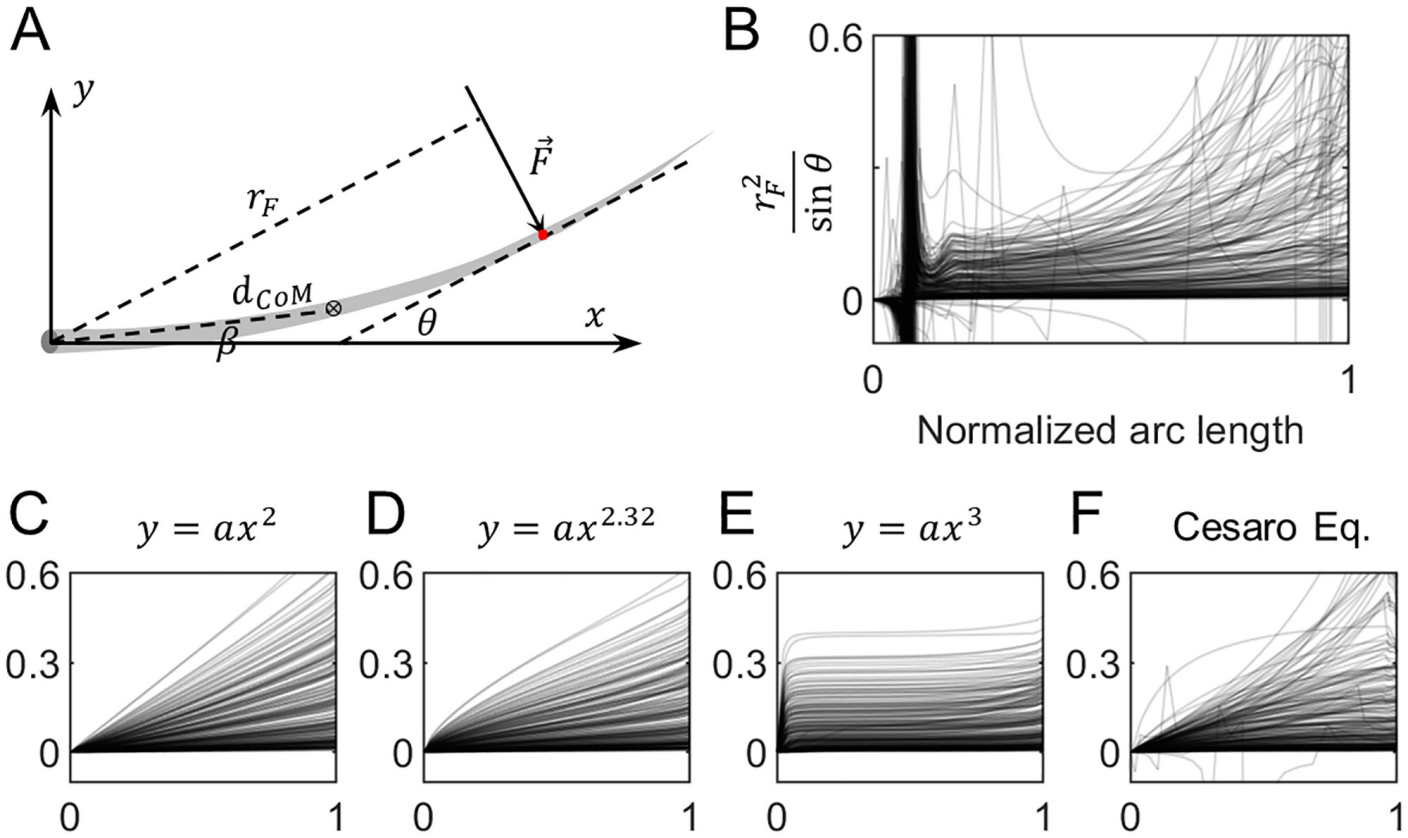
The influence of curvature model on mechanical analysis. **(A)** A whisker reference frame is defined with the whisker in standard orientation. The whisker is allowed to rotate about the z-axis at its base upon contact. The center of mass (CoM) of the whisker is β degrees from the x-axis, with a straight-line distance d_CoM_ to the whisker base. The tangent at the point of contact (red) forms an angle θ to the x-axis. The whisker model is assumed to be frictionless, so the external force of magnitude F is perpendicular to the tangent at the location of contact, with a distance r_F_ to the whisker base. The total mass (M) and the moment of inertia (I) about the z-axis are also known. **(B)** The second factor of the right side of Eq 10 is evaluated along the whisker arc length for 225 raw whisker shapes. **(C-F)** Each panel has the same axes as (B) but results are shown from using different models for approximating the whisker shape. Note that for all panels B- F, the y-axis is truncated at 0.6 for visual clarity.

For simplicity, we assume rigid body rotation, with frictionless contact and no vibrations. In Fig 9A, a whisker is pushed by an external force F at a given point of contact. At an instant of time immediately after contact (t = 0_+_), the angular acceleration α caused by the external force is

$$\alpha ={\left.\frac{r_{F}F}{I}\right|}_{t=0_{+}}$$ (6)

where I represents the moment of inertia about the z-axis. The angular velocity of the whisker is found by considering a variation of time,

$$\delta \omega =\alpha \delta t=\frac{r_{F}F}{I}\delta t$$ (7)

The magnitude of the x-component of the force at the whisker base that counters the centripetal force is

$$F_{\text{centripetal}}=M\delta \omega^{2}d_{CoM}\;\text{cos}\;\beta =\;M{\left(\frac{r_{F}F}{I}\delta t\right)}^{2}d_{CoM}\;\text{cos}\;\beta$$ (8)

while at the same time, the external force applied in the axial direction is

$$F_{\text{external}}=F\;\text{sin}\;\theta$$ (9)

The ratio of these two forces can be divided into two factors, the second of which depends directly on the location of the point of contact

$$\frac{F_{\text{centripetal}}}{F_{\text{external}}}=\frac{MFd_{CoM}\;\text{cos}\;\beta \delta t^{2}}{I^{2}}\cdot \frac{r_{F}^{2}}{\text{sin}\;\theta }$$ (10)

Fig 9B-9F show, for 225 rat whiskers, how the second factor in Eq 10 varies along the whisker arc length. Each panel assumes a different model of curvature, and the curves in each panel are remarkably different from each other.

In particular, notice that using the raw whisker points (Fig 9B) or the Cesàro fit (Fig 9F) yields large spikes near the base: these artifacts occur because discrete points in the most proximal region of the whisker fluctuate around the zero line. This effect also demonstrates that it is not practical to use the “raw shape” of the whisker in mechanical analysis and it is more appropriate to use a model. In addition, the raw whiskers show a larger variability in the forces near the tip, both in magnitude as well as in slope.

## Discussion

### Advantages and disadvantages of different models for intrinsic curvature

The present work has compared and contrasted different approaches for quantifying the intrinsic curvature of whiskers. Different approaches may be appropriate for answering different questions.

The advantage of a single-parameter polynomial model is that it is simple and extremely intuitive: it is easy to visualize a quadratic or a cubic curve. Single parameter polynomial models generate reasonably good fits for all whiskers (Fig 2). The two-parameter polynomial model provides excellent fits for all species. However, a significant disadvantage of both one- and two-parameter polynomial models is that it is not clear how to compare them across species.

The elliptical model (two parameters) does not appear to be a good choice to describe whisker shape. Although it can fit “backwards” whiskers, the residual values are high.

Fig 8 suggests that the fractional exponent model, the two-parameter polynomial model, and the Cesàro equation show promise for quantifying whisker shape.

The fractional exponent model fits whiskers with low residuals, and in addition, it is a single-parameter model that can be shared across species. The fractional exponent approach is the only one that directly yields a single species-specific parameter (β) that can be compared across species. The model coefficient (a_β_) then uniquely identifies each whisker and can be used to quantify differences within the array of a single species.

The two-parameter polynomial model generally yielded the best fits for all whiskers. However, when comparing across whiskers, it is unclear how the two model coefficients are correlated and what their effect is upon the whisker shape. It remains a good approach to simply extract precise whisker shape.

The Cesàro equation (also two parameters) generally yielded the second-best fits for all whiskers, and can also describe backwards whiskers. The Cesàro equation directly describes how curvature changes along the whisker length. Although it is true that any curve described by a Cesàro equation can be transformed to fit on the universal Euler spiral, it is not immediately clear what insights can be obtained by that transformation. A more significant advantage of the Cesàro fit may be that its parameters could represent how the whisker grows as a result of protein synthesis, an idea elaborated further below.

### Choice of RSS as a metric for goodness-of-fit for model selection

We considered several other metrics for model selection before deciding on RSS.

The most standard approach for model selection involves the use of Student’s t-statistics. However, this metric was not appropriate because fitting a whisker, which consists of smooth, equally-spaced nodes, is very different than fitting a collection of noisy data points. For example, we tried using the model y = a_7_x^7^+ a_6_x^6^+ a_5_x^5^+ a_4_x^4^+ a_3_x^3^+ a_2_x^2^ to fit 225 rat whiskers. Results showed that most whiskers had significant p-values (<0.05) up to the 7^th^ order coefficient (S3 Fig). In general, across all species, we found that t-tests for polynomial fits often generated “statistically significant” coefficients up to 10^th^ order and beyond. Such a high order fit may accurately capture the individual shape of a single whisker, but fails as an attempt to generalize the overall shape of many whiskers.

Second, we considered and quickly rejected using a cross-validation approach. In this approach, curves would be fit to a subset of whiskers of each species, and then tested on the remainder of the whiskers of that species. However, because the whiskers have different shapes, they produce different coefficients for the same model, resulting in a family of curves. This renders challenges in cross-validation.

Third, we tried using the skewness of a histogram of the signed residuals as a metric to judge the goodness-of-fit for a family of curves. As described for the fractional exponent model, a fair model is considered to be one that produces a balanced (i.e., not skewed) histogram. However, the statistical skewness (normalized third central moment) was found to be too biased towards the two tails of the dataset. Establishing an appropriate threshold to use with a non-standard metric was challenging and subjective.

Thus, given the choice between a non-standard skewness metric and the more standard RSS metric, we ultimately chose to use RSS. Although RSS would not be a good metric for goodness-of-fit for a standard statistics problem with noisy data, it yields a close match to human estimates for fits to a family of smooth whiskers of a variety of shapes. Notably, RSS decreases as the number of parameters increases. Therefore, it is important to note that a subjective threshold was used to determine whether a higher order term should be added to the polynomial fit, based on human judgment of the trade-off between model simplicity and fit quality (Fig 2).

### Independence of samples

An issue related to goodness-of-fit is that of sample independence. Samples from a single animal are clearly not independent from each other as their shape and size vary systematically across the array [27,41]. In addition, whiskers from each individual within a species may be very different from each other. To address these issues, S4–S9 Figs shows the distribution of model coefficients for all whiskers of all individuals within each species. For different models used, the within-species differences (both median and distribution profile of coefficients) are generally smaller than between-species differences, with a few exceptions between closely-related rodent species. For the sea lion, differences within species could be a result of age (one individual is a sub-adult), or could simply reflect individual variation.

### Whisker curvature as a result of protein synthesis

Given that both whisker taper and intrinsic curvature are formed through keratinization [42], and that whiskers grow from base to tip, it is possible that the A and B coefficients of the Cesàro fits represent individual whisker growth. Although few studies focus on the synthesis of animal whiskers, some insight can be gained from studies of human hair.

Studies on the human hair keratinization process have shown that curliness is programmed from the hair bulb and is linked to asymmetry in differentiation programs [43]. Two major theories have been put forth to describe the mechanism of bending: the segmented keratinization mechanism [44] and single fiber curvature [45–47]. Both theories are based on asymmetric growth of the hair cell layers on different sides of the follicle. Slower hair synthesis on one side causes the hair to bend concave towards that side. The twist in human hair, and also in some mammalian hairs such as wool (but not typically in whiskers), can be explained by the inner root sheath rotation mechanism [48].

Compared to human hair, the shape of which is often quite complicated, whiskers are relatively simple: they do not twist and they do not “kink,” a discontinuity in curvature associated with some human hair. Throughout the lifetime of a whisker, differences in the growth rates at opposite sides of the follicle are reflected as the curvature at different locations along the whisker length. Thus, assuming that the shape of a whisker does not change after emerging from the follicle, the fact that whiskers can be fit by a Cesàro equation suggests that the difference in growth rates on the two sides of the follicle changes linearly with the whisker life cycle.

### Effect of whisker curvature on tactile sensation

Whisker curvature will affect both geometric and mechanical characteristics of active sensing behaviors. In particular, the “sensing volume,” defined as the volume encompassed by the surface interpolated from all whisker tips [28,49], is directly affected by whisker arc length and intrinsic curvature. The sensing volume determines the spatiotemporal characteristics of the search space during active whisking, and thus the intrinsic curvature could directly affect at least three behaviors. First, the intrinsic curvature works with taper to control the “preview distance,” allowing an effective mapping of body-to-wall distance [50], as well as guiding paw placement during locomotion [51]. Second, a specific sensing volume generated by whiskers of particular shapes will alter the timings within a contact sequence as the whiskers sweep over an object [52]. Third, given the diversity in whisker basepoint locations, the intrinsic curvature of the whiskers could align the tips around the mouth to facilitate prey capture [49].

A straight whisker also lacks multiple mechanical sensing features inherent to a curved whisker. Most obviously, a curved whisker has different slopes along its length, directly altering the components of axial and transverse force [5,6,17,36,53]. The center of mass of a whisker with high curvature will lie at a different location than a straight whisker, which can have nonlinear effects on the magnitude of the signals generated during non-contact whisking [35,36,54]. Finally, a curved whisker helps reduce force focusing and the chances of buckling if contacts occur near the whisker tip.

The intrinsic curvature is not expected to greatly change the resonant frequency modes of the whisker, and thus may have minimal effects on the analysis of vibrations and texture exploration. However, it is well known that whiskers are important for fluid sensing, both for rats [12] and harbor seals [55–58]. In this case, additional non-linear whisker curvature may permit coupled-mode aeroelastic flutter to occur [13,59].

In the analysis of thin beam mechanics, taper has a larger influence on bending than does curvature, therefore previous studies have primarily modeled whiskers and whisker-like structures as straight tapered cones (e.g., [24,33,60,61]. The gradual taper of a whisker means that it is more flexible in its distal regions (towards the tip). During hair growth, it is possible that changes in taper and curvature are influenced by a common governing factor, and it is also possible that the increased flexibility associated with taper gradually and subtly changes the whisker’s intrinsic curvature. Given that both whisker taper and curvature vary systematically across the whisker array [15,20–24,41], it is tempting to postulate that these two parameters may be correlated, not only at the level of a single whisker but also across the array.

## Supporting information

S1 Fig A sampling of whiskers shows whisker planarity. For each animal, ten longer (caudal) whiskers were chosen randomly for careful measurement. Whiskers of harbor seals and sea lions were excluded because they dehydrate rapidly after scanning and do not maintain their shape. Whisker identities were indexed in a similar way to rat whiskers (side, row, column). R: Right. L: Left. A-E: Row number, from dorsal to ventral. 01–03: Column number, from the most caudal to rostral. On average, 90.6% of the full length of the sampled whisker is planar.
(PDF)

S2 Fig Examples of planar and non-planar whiskers. Each panel shows the front view (top) and birds-eye view (bottom) of a whisker. The whisker tips are only slightly out of the plane, which has almost no effect on its 2D projection. No twist is observed in any of the whisker shape.
(PDF)

S3 Fig Student’s t-statistics show rat whiskers can be fit by a polynomial model of at least 7^th^ order. 225 rat whiskers were fit to the model y = a_7_x^7^+a_6_x^6^+a_5_x^5^+a_4_x^4^+a_3_x^3^+a_2_x^2^. The p-values generated for the coefficient of each term are mostly smaller than 0.05, showing all terms are significant in fitting a whisker.
(PDF)

S4 Fig Individual variation in the polynomial model y = a_2_x^2^. The optimized model coefficients a_2_ for each whisker are plotted as violin plots grouped by individual animals. The x-axis of each plot shows data for each individual of that species. The probability distribution of the exponent value for each animal is indicates by the “violin” at that location, i.e., the width of the violin represents the fraction of whiskers with that coefficient value. Black horizontal bar indicates the mean. Red horizontal bar indicates the median.
(PDF)

S5 Fig Individual variation in the polynomial model y = a_3_x^3^. The optimized model coefficients a_3_ for each whisker are plotted as violin plots grouped by individual animals. Plotting conventions are identical to S4 Fig.
(PDF)

S6 Fig Individual variation in the fractional exponent model y = a_β_x^β^. The optimized model coefficients a_β_ for each whisker are plotted as violin plots grouped by individual animals. It is important to keep in mind that each subplot (i.e., each species) has a different value of β (top right corner). Plotting conventions are identical to S4 Fig.
(PDF)

S7 Fig Individual variation in the polynomial model y = a_3_x^3^+a_2_x^2^. The optimized model coefficients, a_3_ and a_2_, are presented in a 2D scatter plot. The marginal probability density distributions of different species are shown below and to the right of the main panel. Statistics for individuals of the same species are indicated as stacked solid and dashed lines. Each solid line represents statistics for one individual animal and extends from the lower to the upper quartile. Dashed lines extend further, to the minimum and maximum values for each individual, excluding outliers.
(PDF)

S8 Fig Individual variation in the elliptical model. The optimized coefficients for the elliptical model, A and B, are presented in a 2D scatter plot. Plotting conventions are identical to S7 Fig.
(PDF)

S9 Fig Individual variation in the linear Cesàro model. The optimized coefficients A and B in the linear Cesàro equation κ(s) = As+B, are presented in a 2D scatter plot. Plotting conventions are identical to S7 Fig.
(PDF)

## References

[1] Grant RA, Sperber AL, Prescott TJ. The role of orienting in vibrissal touch sensing. Front Behav Neurosci. 2012;6:39. doi: 10.3389/fnbeh.2012.00039 22787445 PMC3391677

[2] Grant RA, Mitchinson B, Prescott TJ. The development of whisker control in rats in relation to locomotion. Developmental Psychobiology. 2012;54(2):151–68. doi: 10.1002/dev.20591 22231841

[3] Munz M, Brecht M, Wolfe J. Active touch during shrew prey capture. Front Behav Neurosci. 2010;4:191. doi: 10.3389/fnbeh.2010.00191 21283557 PMC3028568

[4] Muchlinski MN. A comparative analysis of vibrissa count and infraorbital foramen area in primates and other mammals. Journal of Human Evolution. 2010;58(6):447–73. doi: 10.1016/j.jhevol.2010.01.012 20434193

[5] Quist BW, Hartmann MJZ. Mechanical signals at the base of a rat vibrissa: the effect of intrinsic vibrissa curvature and implications for tactile exploration. J Neurophysiol. 2012;107(9):2298–312. doi: 10.1152/jn.00372.2011 22298834 PMC3362248

[6] Yang AE, Hartmann MJ. Whisking kinematics enables object localization in head-centered coordinates based on tactile information from a single vibrissa. Front Behavioral Neuroscience. 2016;10:145. doi: 10.3389/fnbeh.2016.00145 27486390 PMC4949211

[7] Whiteley SJ, Knutsen PM, Matthews DW, Kleinfeld D. Deflection of a vibrissa leads to a gradient of strain across mechanoreceptors in a mystacial follicle. J Neurophysiol. 2015;114(1):138–45. doi: 10.1152/jn.00179.2015 25855692 PMC4507969

[8] Luo Y, Bresee CS, Rudnicki JW, Hartmann MJZ. Constraints on the deformation of the vibrissa within the follicle. PLoS Comput Biol. 2021;17(4):e1007887. doi: 10.1371/journal.pcbi.1007887 33793548 PMC8016108

[9] Ebara S, Kumamoto K, Matsuura T, Mazurkiewicz JE, Rice FL. Similarities and differences in the innervation of mystacial vibrissal follicle-sinus complexes in the rat and cat: a confocal microscopic study. J Comp Neurol. 2002;449(2):103–19. doi: 10.1002/cne.10277 12115682

[10] Furuta T, Bush NE, Yang AE, Ebara S, Miyazaki N, Murata K, et al. The cellular and mechanical basis for response characteristics of identified primary afferents in the rat vibrissal system. Curr Biol. 2020. doi: 10.1016/j.cub.2019.12.068 32004452 PMC10623402

[11] Hipp J, Arabzadeh E, Zorzin E, Conradt J, Kayser C, Diamond ME, et al. Texture signals in whisker vibrations. J Neurophysiol. 2006;95(3):1792–9. doi: 10.1152/jn.01104.2005 16338992

[12] Yu YSW, Graff MM, Bresee CS, Man YB, Hartmann MJZ. Whiskers aid anemotaxis in rats. Sci Adv. 2016;2(8):e1600716. doi: 10.1126/sciadv.1600716 27574705 PMC4996642

[13] Yu YS, Graff MM, Hartmann MJ. Mechanical responses of rat vibrissae to airflow. J Exp Biol. 2016;219(Pt 7):937–48. doi: 10.1242/jeb.126896 27030774 PMC4852692

[14] Pammer L, O’Connor DH, Hires SA, Clack NG, Huber D, Myers EW, et al. The mechanical variables underlying object localization along the axis of the whisker. J Neurosci. 2013;33(16):6726–41. doi: 10.1523/JNEUROSCI.4316-12.2013 23595731 PMC3733083

[15] Williams CM, Kramer EM. The advantages of a tapered whisker. PLoS One. 2010;5(1):e8806. doi: 10.1371/journal.pone.0008806 20098714 PMC2808387

[16] Quist BW, Faruqi RA, Hartmann MJZ. Variation in Young’s modulus along the length of a rat vibrissa. J Biomech. 2011;44(16):2775–81. doi: 10.1016/j.jbiomech.2011.08.027 21993474

[17] Solomon JH, Hartmann MJ. Radial distance determination in the rat vibrissal system and the effects of Weber’s law. Philos Trans R Soc Lond B Biol Sci. 2011;366(1581):3049–57. doi: 10.1098/rstb.2011.0166 21969686 PMC3172605

[18] Huet LA, Rudnicki JW, Hartmann MJZ. Tactile Sensing with Whiskers of Various Shapes: Determining the Three-Dimensional Location of Object Contact Based on Mechanical Signals at the Whisker Base. Soft Robot. 2017;4(2):88–102. doi: 10.1089/soro.2016.0028 28616371 PMC5467137

[19] Schultz AE, Solomon JH, Peshkin MA, Hartmann MJ, editors. Multifunctional whisker arrays for distance detection, terrain mapping, and object feature extraction. Proceedings of the 2005 IEEE International Conference on Robotics and Automation; 2005; Barcelona, Spain.

[20] Ibrahim L, Wright EA. The growth of rats and mice vibrissae under normal and some abnormal conditions. Development. 1975;33(4):831–44. 1176877

[21] Boubenec Y, Claverie LN, Shulz DE, Debregeas G. An amplitude modulation/demodulation scheme for whisker-based texture perception. J Neurosci. 2014;34(33):10832–43. doi: 10.1523/JNEUROSCI.0534-14.2014 25122886 PMC6705261

[22] Carl K, Hild W, Mampel J, Schilling C, Uhlig R, Witte H. Characterization of statical properties of rat’s whisker system. IEEE Sens J. 2012;12(2):340–9.

[23] Voges D, Carl K, Klauer GJ, Uhlig R, Schilling C, Behn C, et al. Structural Characterization of the Whisker System of the Rat. IEEE Sens J. 2012;12(2):332–9.

[24] Hires SA, Schuyler A, Sy J, Huang V, Wyche I, Wang X, et al. Beyond cones: an improved model of whisker bending based on measured mechanics and tapering. J Neurophysiol. 2016;116(2):812–24. doi: 10.1152/jn.00511.2015 27250911 PMC4995282

[25] Knutsen PM, Biess A, Ahissar E. Vibrissal kinematics in 3D: Tight coupling of azimuth, elevation, and torsion across different whisking modes. Neuron. 2008;59(1):35–42. doi: 10.1016/j.neuron.2008.05.013 18614027

[26] Towal RB, Quist BW, Gopal V, Solomon JH, Hartmann MJZ. The morphology of the rat vibrissal array: A model for quantifying spatiotemporal patterns of whisker-object contact. PLoS Comput Biol. 2011;7(4):e1001120. doi: 10.1371/journal.pcbi.1001120 21490724 PMC3072363

[27] Belli HM, Bresee CS, Graff MM, Hartmann MJZ. Quantifying the three-dimensional facial morphology of the laboratory rat with a focus on the vibrissae. Plos One. 2018;13(4):31. doi: 10.1371/journal.pone.0194981 29621356 PMC5886528

[28] Starostin EL, Grant RA, Dougill G, van der Heijden GHM, Goss VGA. The Euler spiral of rat whiskers. Sci Adv. 2020;6(3):eaax5145. doi: 10.1126/sciadv.aax5145 31998835 PMC6962041

[29] Lucianna FA, Albarracin AL, Vrech SM, Farfan FD, Felice CJ. The mathematical whisker: A review of numerical models of the rat’s vibrissa biomechanics. J Biomech. 2016;49(10):2007–14. 27260019 10.1016/j.jbiomech.2016.05.019

[30] Dougill G, Starostin EL, Milne AO, van der Heijden GHM, Goss VGA, Grant RA. Ecomorphology reveals Euler spiral of mammalian whiskers. J Morphol. 2020;281(10):1271–9. doi: 10.1002/jmor.21246 32738083

[31] Campagner D, Evans MH, Loft MSE, Petersen RS. What the whiskers tell the brain. Neuroscience. 2018;368:95–108. doi: 10.1016/j.neuroscience.2017.08.005 28843998

[32] Knutsen PM, Derdikman D, Ahissar E. Tracking whisker and head movements in unrestrained behaving rodents. J Neurophysiol. 2005;93(4):2294–301. doi: 10.1152/jn.00718.2004 15563552

[33] Solomon JH, Hartmann MJ. Biomechanics: robotic whiskers used to sense features. Nature. 2006;443(7111):525. doi: 10.1038/443525a 17024083

[34] Birdwell JA, Solomon JH, Thajchayapong M, Taylor MA, Cheely M, Towal RB, et al. Biomechanical models for radial distance determination by the rat vibrissal system. J Neurophysiol. 2007;98(4):2439–55. doi: 10.1152/jn.00707.2006 17553946

[35] Quist BW, Seghete V, Huet LA, Murphey TD, Hartmann MJZ. Modeling forces and moments at the base of a rat vibrissa during noncontact whisking and whisking against an object. J Neurosci. 2014;34(30):9828–44. doi: 10.1523/JNEUROSCI.1707-12.2014 25057187 PMC4107402

[36] Campagner D, Evans MH, Bale MR, Erskine A, Petersen RS. Prediction of primary somatosensory neuron activity during active tactile exploration. eLife. 2016;5. doi: 10.7554/eLife.10696 26880559 PMC4764568

[37] Bush NE, Solla SA, Hartmann MJZ. Whisking mechanics and active sensing. Curr Opin Neurobiol. 2016;40:178–88. doi: 10.1016/j.conb.2016.08.001 27632212 PMC5312677

[38] Zweifel NO, Bush NE, Abraham I, Murphey TD, Hartmann MJZ. A dynamical model for generating synthetic data to quantify active tactile sensing behavior in the rat. Proc Natl Acad Sci U S A. 2021;118(27). doi: 10.1073/pnas.2011905118 34210794 PMC8271597

[39] Hires SA, Pammer L, Svoboda K, Golomb D. Tapered whiskers are required for active tactile sensation. eLife. 2013;2:doi: 10.7554/eLife.01350 24252879 PMC3828597

[40] O’Connor DH, Clack NG, Huber D, Komiyama T, Myers EW, Svoboda K. Vibrissa-based object localization in head-fixed mice. J Neurosci. 2010;30(5):1947–67. doi: 10.1523/JNEUROSCI.3762-09.2010 20130203 PMC6634009

[41] Belli HM, Yang AE, Bresee CS, Hartmann MJZ. Variations in vibrissal geometry across the rat mystacial pad: base diameter, medulla, and taper. J Neurophysiol. 2017;117(4):1807–20. doi: 10.1152/jn.00054.2016 27881718 PMC5390285

[42] Chase HB. Growth of the hair. Physiological Reviews. 1954;34(1):113–26. doi: 10.1152/physrev.1954.34.1.113 13120379

[43] Thibaut S, Gaillard O, Bouhanna P, Cannell DW, Bernard BA. Human hair shape is programmed from the bulb. Br J Dermatol. 2005;152(4):632–8. doi: 10.1111/j.1365-2133.2005.06521.x 15840091

[44] Kassenbeck P. Morphology and fine structure of hair. Hair Research; Berlin, Heidelberg 1981. p. 52–64.

[45] Dobozy OK. The shape and cause of wool crimp. Textile research journal. 1959;29(10):836–9.

[46] Brown TD, Onions WJ. A Theory for the Development of Wool Fibre Crimp on Drying. Journal of the Textile Institute Transactions. 1961;52(3):T101–T8.

[47] Bryson WG, Harland DP, Caldwell JP, Vernon JA, Walls RJ, Woods JL, et al. Cortical cell types and intermediate filament arrangements correlate with fiber curvature in Japanese human hair. J Struct Biol. 2009;166(1):46–58. doi: 10.1016/j.jsb.2008.12.006 19159689

[48] Nagorcka PN. Theoretical mechanism for crimp. Australian Journal of Biological Sciences. 1981;34(2):189–210.

[49] Huet LA, Hartmann MJ. The search space of the rat during whisking behavior. J Exp Biol. 2014;217(Pt 18):3365–76. doi: 10.1242/jeb.105338 25232200

[50] Mongeau JM, Demir A, Dallmann CJ, Jayaram K, Cowan NJ, Full RJ. Mechanical processing via passive dynamic properties of the cockroach antenna can facilitate control during rapid running. J Exp Biol. 2014;217(Pt 18):3333–45. doi: 10.1242/jeb.101501 25013115

[51] Grant RA, Breakell V, Prescott TJ. Whisker touch sensing guides locomotion in small, quadrupedal mammals. Proc Biol Sci. 2018;285(1880). doi: 10.1098/rspb.2018.0592 29899069 PMC6015872

[52] Mehta SB, Whitmer D, Figueroa R, Williams BA, Kleinfeld D. Active spatial perception in the vibrissa scanning sensorimotor system. PLoS Biol. 2007;5(2):e15. doi: 10.1371/journal.pbio.0050015 17227143 PMC1769422

[53] Bagdasarian K, Szwed M, Knutsen PM, Deutsch D, Derdikman D, Pietr M, et al. Pre-neuronal morphological processing of object location by individual whiskers. Nat Neurosci. 2013;16(5):622–31. doi: 10.1038/nn.3378 23563582

[54] Wallach A, Bagdasarian K, Ahissar E. On-going computation of whisking phase by mechanoreceptors. Nat Neurosci. 2016;19(3):487–93. doi: 10.1038/nn.4221 26780508

[55] Dehnhardt G, Mauck B, Hanke W, Bleckmann H. Hydrodynamic trail-following in harbor seals (Phoca vitulina). Science. 2001;293(5527):102–4. doi: 10.1126/science.1060514 11441183

[56] Glaser N, Wieskotten S, Otter C, Dehnhardt G, Hanke W. Hydrodynamic trail following in a California sea lion (Zalophus californianus). Journal of Comparative Physiology A. 2011;197(2):141–51. 10.1007/s00359-010-0594-5 20959994

[57] Miersch L, Hanke W, Wieskotten S, Hanke FD, Oeffner J, Leder A, et al. Flow sensing by pinniped whiskers. Philosophical Transactions of the Royal Society B: Biological Sciences. 2011;366(1581):3077–84. doi: 10.1098/rstb.2011.0155 21969689 PMC3172597

[58] Wieskotten S, Mauck B, Miersch L, Dehnhardt G, Hanke W. Hydrodynamic discrimination of wakes caused by objects of different size or shape in a harbour seal (Phoca vitulina). J Exp Biol. 2011;214(11):1922–30. doi: 10.1242/jeb.053926 21562180

[59] Heydari S, Patankar N, Hartmann MJZ, Jaiman R. Self-excited aeroelastic instability of a flexible cantilever cylinder at laminar subcritical Reynolds number. American Physical Society 2021.

[60] Kim D, Möller R. Biomimetic whiskers for shape recognition. Robotics and Autonomous Systems. 2007;55(3):229–43.

[61] Bebek O, Cavusoglu MC. Whisker-like position sensor for measuring physiological motion. IEEE/ASME Transactions on Mechatronics. 2008;13(5):538–47.

