# Supplementary material for: On the intrinsic curvature of animal whiskers
Source: PLoS One. 2023 Jan 6;18(1):e0269210. doi: 10.1371/journal.pone.0269210 (PMC9821693; doi:10.1371/journal.pone.0269210)
Supplement: S1 Fig — For each animal, ten longer (caudal) whiskers were chosen randomly for careful measurement. Whiskers of harbor seals and sea lions were excluded because they dehydrate rapidly after scanning and do not maintain their shape. Whisker identities were indexed in a similar way to rat whiskers (side, row, column). R: Right. L: Left. A-E: Row number, from dorsal to ventral. 01–03: Column number, from the most caudal to rostral. On average, 90.6% of the full length of the sampled whisker is planar. (PDF) [file pone.0269210.s001.pdf]

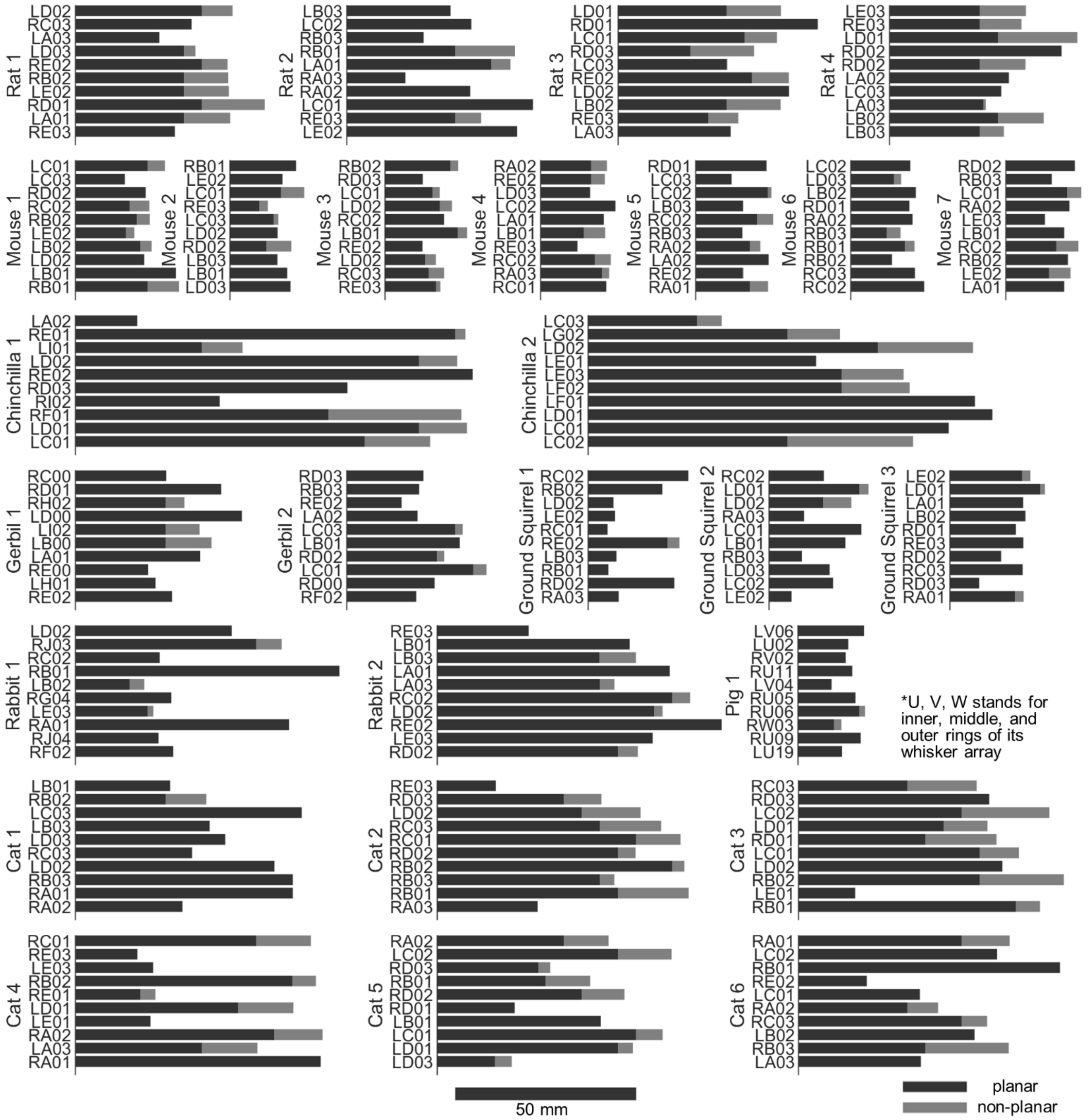

S1 Fig. A sampling of whiskers shows whisker planarity. For each animal, ten longer (caudal) whiskers were chosen randomly for careful measurement. Whiskers of harbor seals and sea lions were excluded because they dehydrate rapidly after scanning and do not maintain their shape. Whisker identities were indexed in a similar way to rat whiskers (side, row, column). R: right. L: left. A-E: row number, from dorsal to ventral. 01-03: column number, from the most caudal to rostral. On average, 90.6% of the full length of the sampled whisker is planar.
