# Supplementary material for: On the intrinsic curvature of animal whiskers
Source: PLoS One. 2023 Jan 6;18(1):e0269210. doi: 10.1371/journal.pone.0269210 (PMC9821693; doi:10.1371/journal.pone.0269210)
Supplement: S2 Fig — Each panel shows the front view (top) and birds-eye view (bottom) of a whisker. The whisker tips are only slightly out of the plane, which has almost no effect on its 2D projection. No twist is observed in any of the whisker shape. (PDF) [file pone.0269210.s002.pdf]

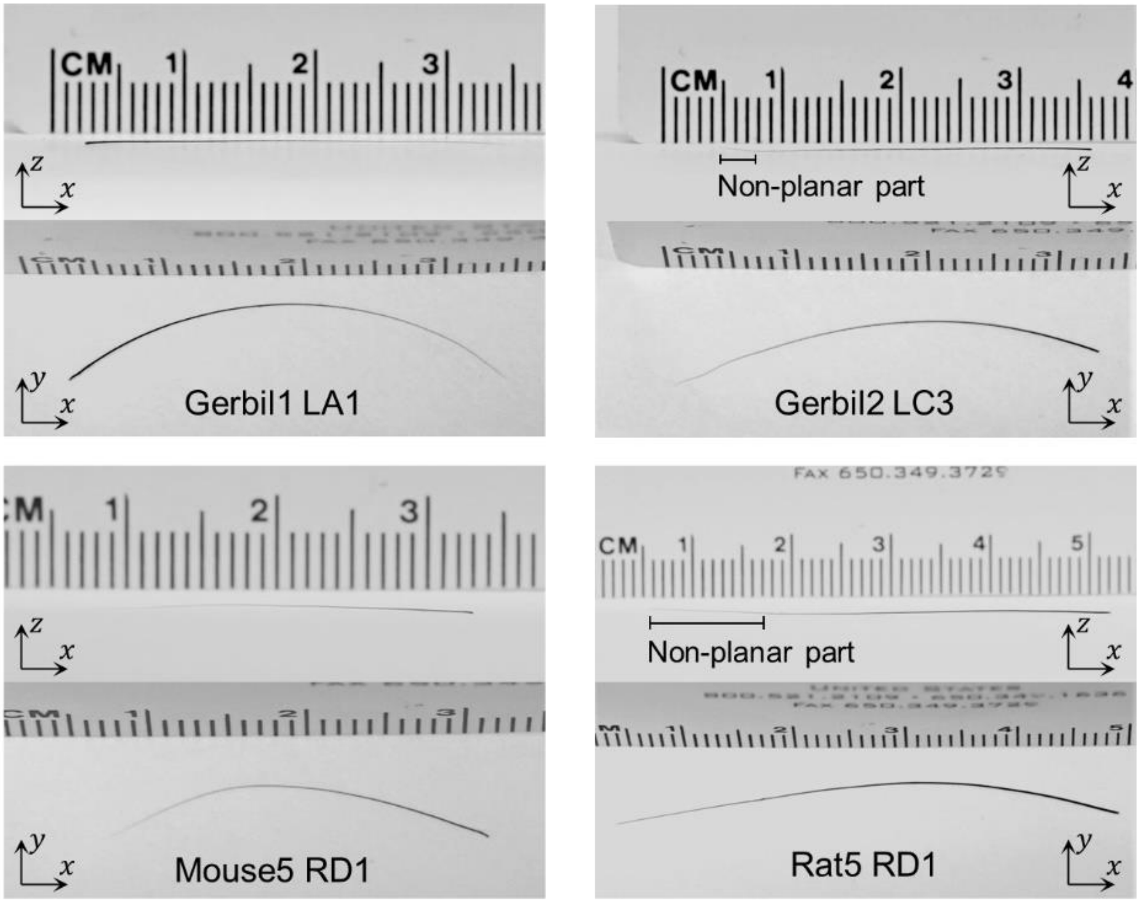

S2 Fig. Examples of planar and non-planar whiskers. Each panel shows the front view (top) and birds-eye view (bottom) of a whisker. The whisker tips are only slightly out of the plane, which has almost no effect on its 2D projection. No twist is observed in any of the whisker shape.
