# Supplementary material for: On the intrinsic curvature of animal whiskers
Source: PLoS One. 2023 Jan 6;18(1):e0269210. doi: 10.1371/journal.pone.0269210 (PMC9821693; doi:10.1371/journal.pone.0269210)
Supplement: S3 Fig — 225 rat whiskers were fit to the model y = a7x7+a6x6+a5x5+a4x4+a3x3+a2x2. The p-values generated for the coefficient of each term are mostly smaller than 0.05, showing all terms are significant in fitting a whisker. (PDF) [file pone.0269210.s003.pdf]

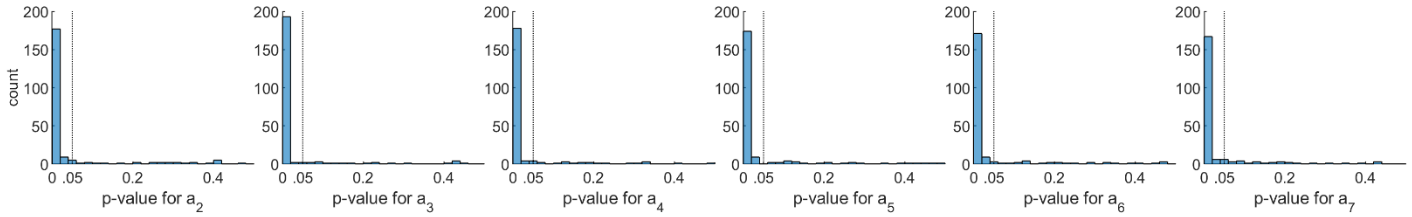

S3 Fig. Student's t-statistics show rat whiskers can be fit by a polynomial model of at least 7th order. 225 rat whiskers were fit to the model $y=a_7x^7+a_6x^6+a_5x^5+a_4x^4+a_3x^3+a_2x^2$. The p-values generated for the coefficient of each term are mostly smaller than 0.05, showing all terms are significant in fitting a whisker.
