# Supplementary material for: On the intrinsic curvature of animal whiskers
Source: PLoS One. 2023 Jan 6;18(1):e0269210. doi: 10.1371/journal.pone.0269210 (PMC9821693; doi:10.1371/journal.pone.0269210)
Supplement: S4 Fig — The optimized model coefficients a2 for each whisker are plotted as violin plots grouped by individual animals. The x-axis of each plot shows data for each individual of that species. The probability distribution of the exponent value for each animal is indicates by the “violin” at that location, i.e., the width of the violin represents the fraction of whiskers with that coefficient value. Black horizontal bar indicates the mean. Red horizontal bar indicates the median. (PDF) [file pone.0269210.s004.pdf]

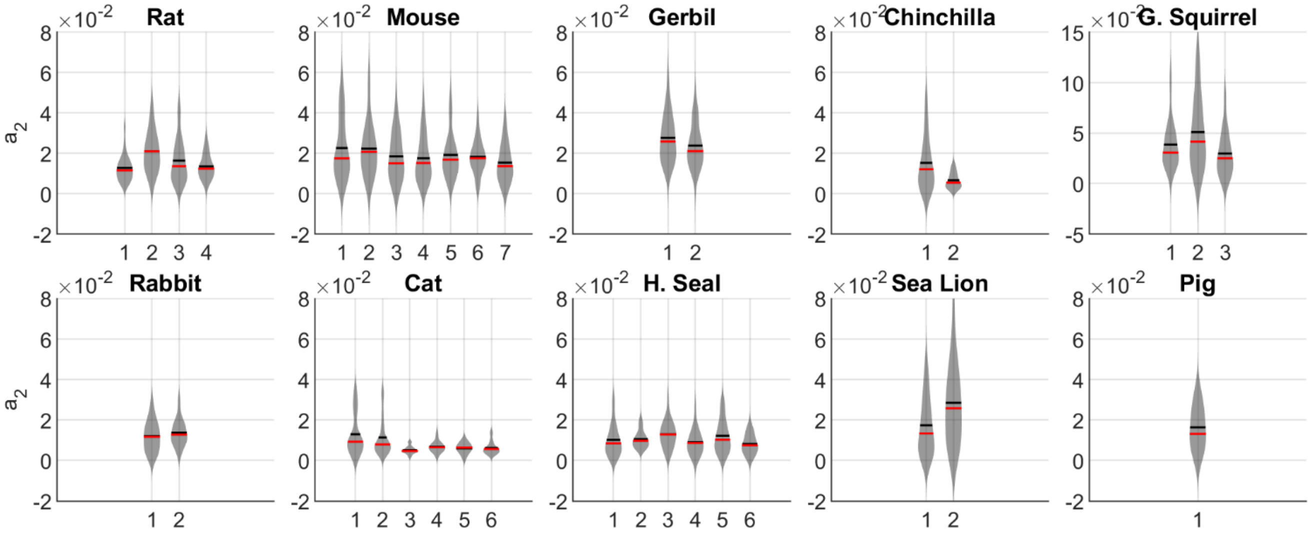

S4 Fig. Individual variation in the polynomial model $y=a_2x^2$. The optimized model coefficients a_2 for each whisker are plotted as violin plots grouped by individual animals. The x-axis of each plot shows data for each individual of that species. The probability distribution of the exponent value for each animal is indicates by the "violin" at that location, i.e., the width of the violin represents the fraction of whiskers with that coefficient value. Black horizontal bar indicates the mean. Red horizontal bar indicates the median.
