# Supplementary material for: On the intrinsic curvature of animal whiskers
Source: PLoS One. 2023 Jan 6;18(1):e0269210. doi: 10.1371/journal.pone.0269210 (PMC9821693; doi:10.1371/journal.pone.0269210)
Supplement: S5 Fig — The optimized model coefficients a3 for each whisker are plotted as violin plots grouped by individual animals. Plotting conventions are identical to S4 Fig. (PDF) [file pone.0269210.s005.pdf]

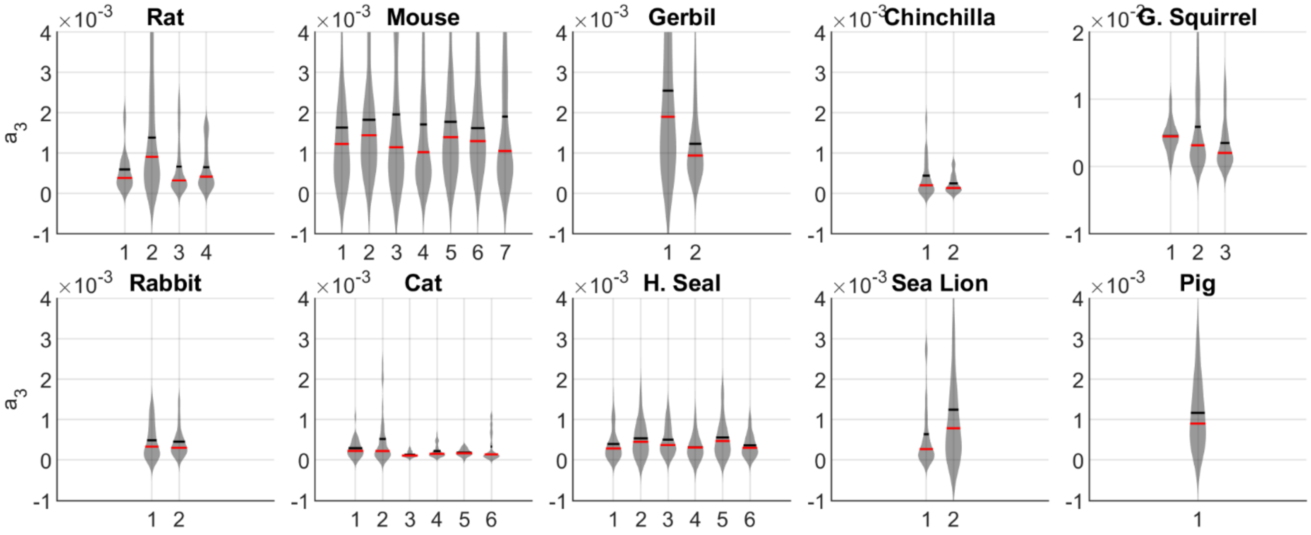

S5 Fig. Individual variation in the polynomial model $y=a_3x^3$. The optimized model coefficients a_3 for each whisker are plotted as violin plots grouped by individual animals. Plotting conventions are identical to S4 Fig.
