# Supplementary material for: On the intrinsic curvature of animal whiskers
Source: PLoS One. 2023 Jan 6;18(1):e0269210. doi: 10.1371/journal.pone.0269210 (PMC9821693; doi:10.1371/journal.pone.0269210)
Supplement: S6 Fig — The optimized model coefficients aβ for each whisker are plotted as violin plots grouped by individual animals. It is important to keep in mind that each subplot (i.e., each species) has a different value of β (top right corner). Plotting conventions are identical to S4 Fig. (PDF) [file pone.0269210.s006.pdf]

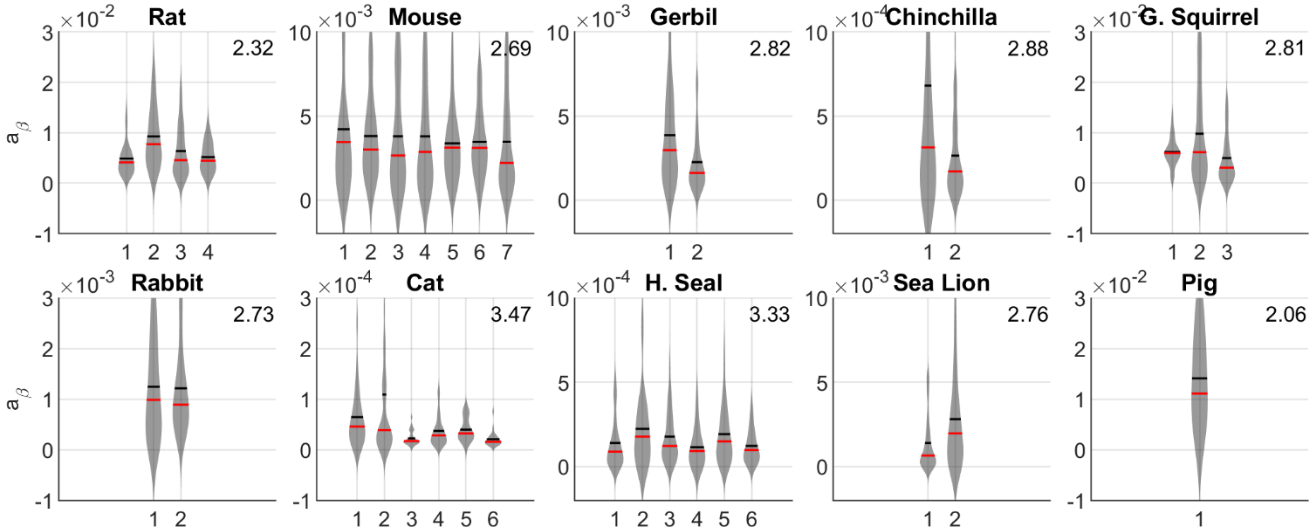

S6 Fig. Individual variation in the fractional exponent model $y=a_\beta x^\beta$. The optimized model coefficients a_β for each whisker are plotted as violin plots grouped by individual animals. It is important to keep in mind that each subplot (i.e., each species) uses a different value of β (top right corner). Plotting conventions are identical to S4 Fig.
