# Supplementary material for: On the intrinsic curvature of animal whiskers
Source: PLoS One. 2023 Jan 6;18(1):e0269210. doi: 10.1371/journal.pone.0269210 (PMC9821693; doi:10.1371/journal.pone.0269210)
Supplement: S7 Fig — The optimized model coefficients, a3 and a2, are presented in a 2D scatter plot. The marginal probability density distributions of different species are shown below and to the right of the main panel. Statistics for individuals of the same species are indicated as stacked solid and dashed lines. Each solid line represents statistics for one individual animal and extends from the lower to the upper quartile. Dashed lines extend further, to the minimum and maximum values for each individual, excluding outliers. (PDF) [file pone.0269210.s007.pdf]

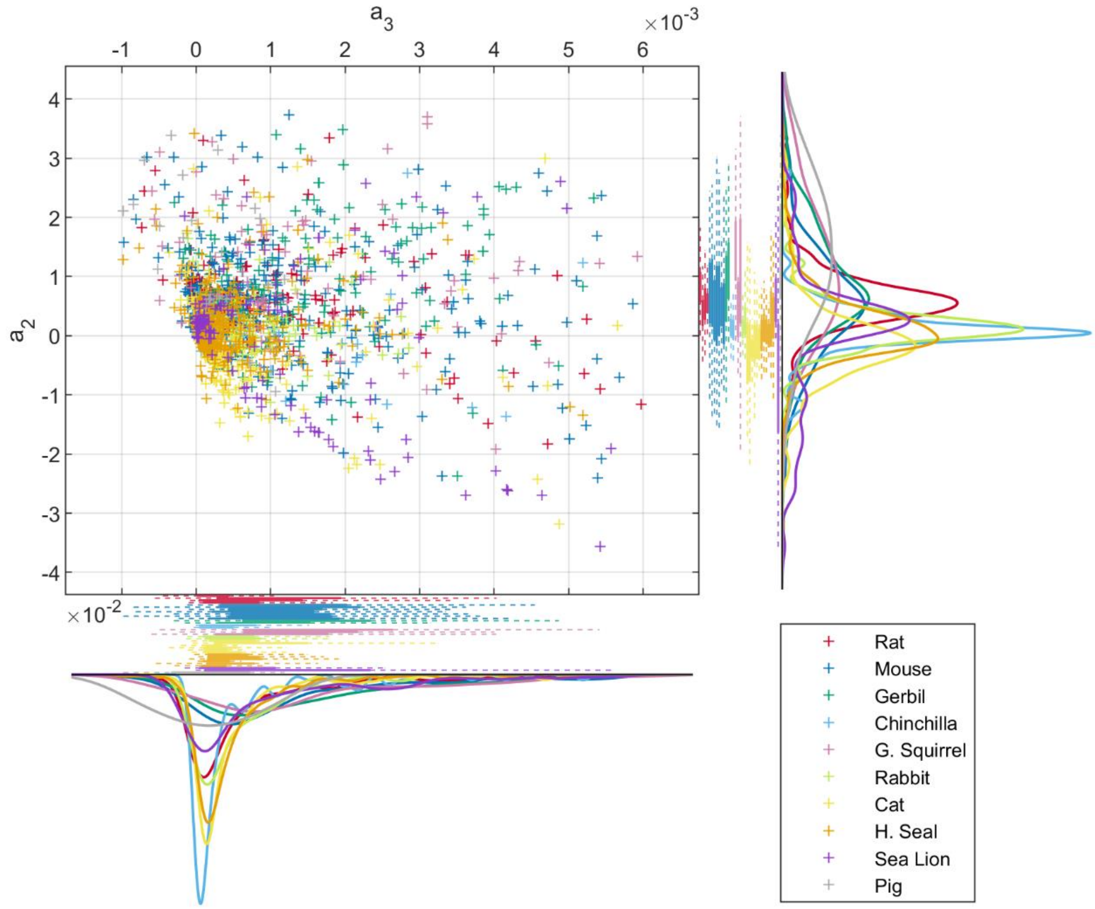

S7 Fig. Individual variation in the polynomial model $y=a_3x^3+a_2x^2$. The optimized model coefficients, a_3 and a_2, are presented in a 2D scatter plot. The marginal probability density distributions of different species are shown below and to the right of the main panel. Statistics for individuals of the same species are indicated as stacked solid and dashed lines. Each solid line represents statistics for one individual animal and extends from the lower to the upper quartile. Dashed lines extend further, to the minimum and maximum values for each individual, excluding outliers.
