# Supplementary material for: On the intrinsic curvature of animal whiskers
Source: PLoS One. 2023 Jan 6;18(1):e0269210. doi: 10.1371/journal.pone.0269210 (PMC9821693; doi:10.1371/journal.pone.0269210)
Supplement: S8 Fig — The optimized coefficients for the elliptical model, A and B, are presented in a 2D scatter plot. Plotting conventions are identical to S7 Fig. (PDF) [file pone.0269210.s008.pdf]

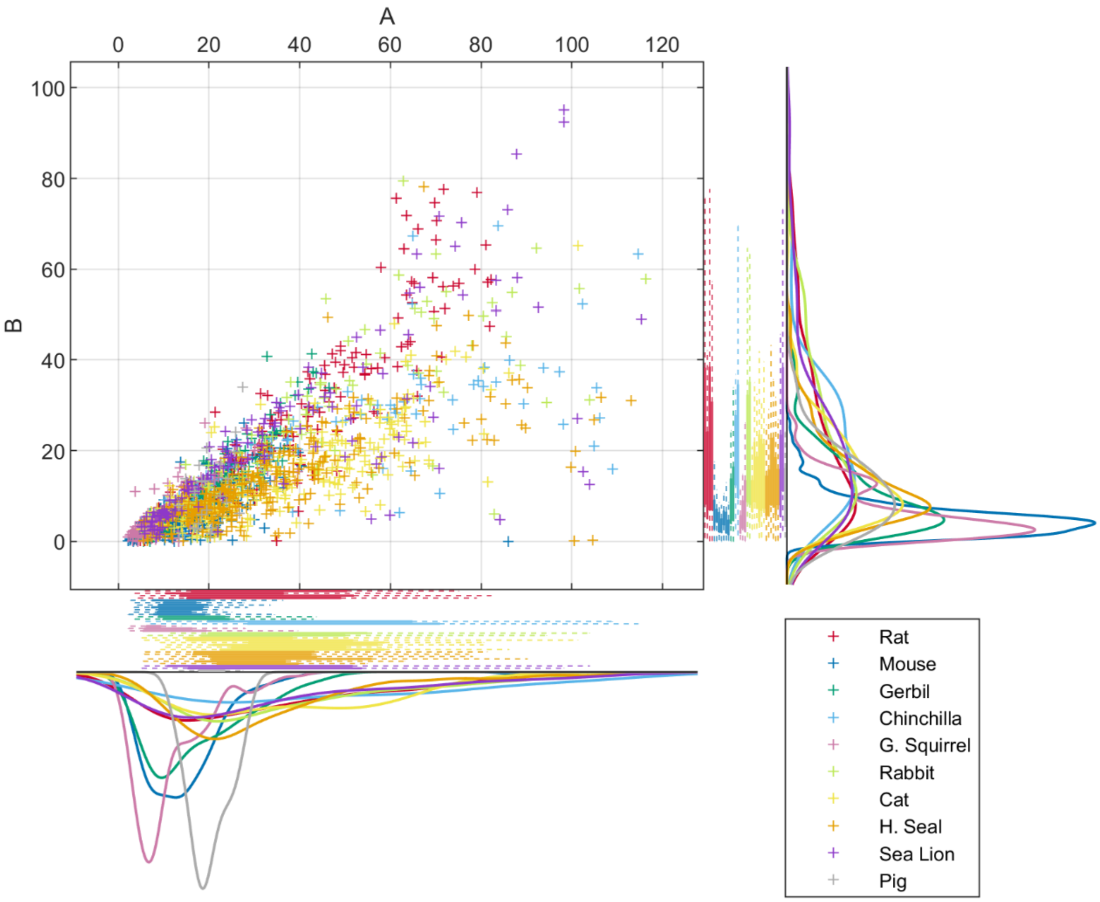

S8 Fig. Individual variation in the elliptical model. The optimized coefficients for the elliptical model, A and B, are presented in a 2D scatter plot. Plotting conventions are identical to S7 Fig.
