# Supplementary material for: On the intrinsic curvature of animal whiskers
Source: PLoS One. 2023 Jan 6;18(1):e0269210. doi: 10.1371/journal.pone.0269210 (PMC9821693; doi:10.1371/journal.pone.0269210)
Supplement: S9 Fig — The optimized coefficients A and B in the linear Cesàro equation κ(s) = As+B, are presented in a 2D scatter plot. Plotting conventions are identical to S7 Fig. (PDF) [file pone.0269210.s009.pdf]

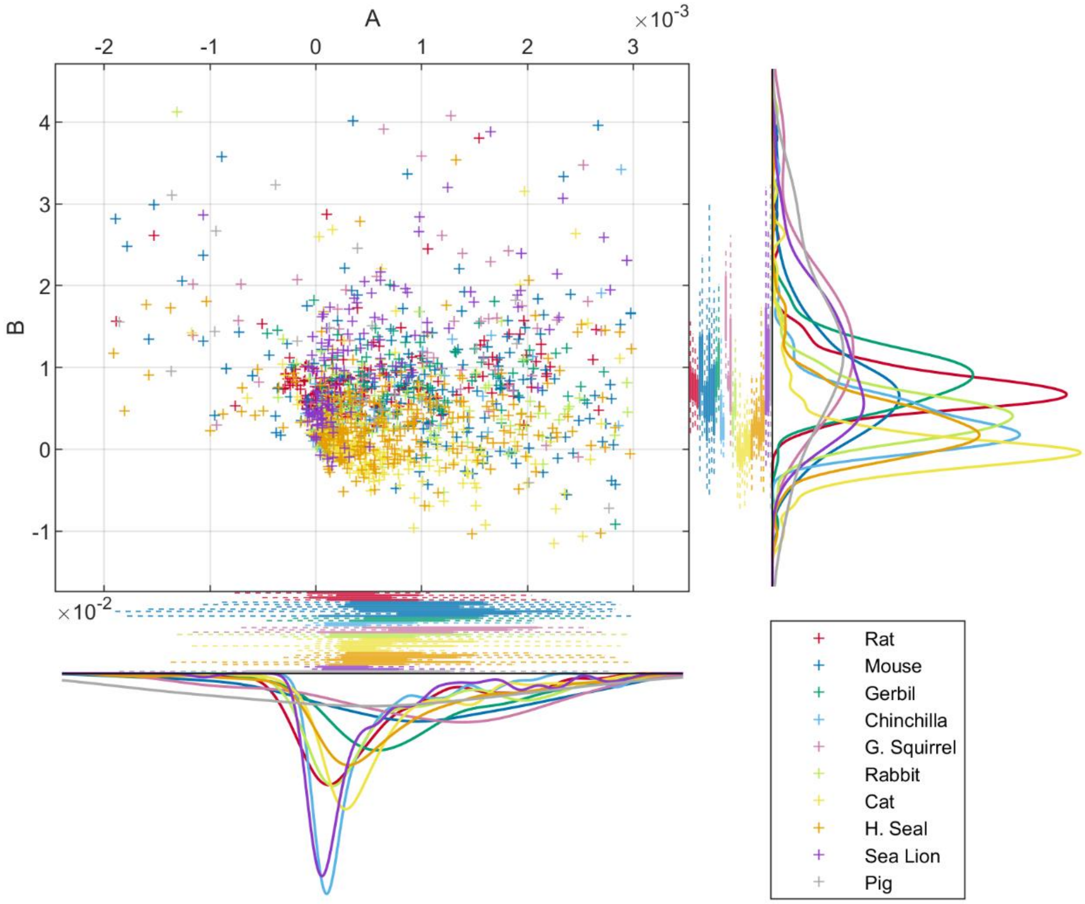

S9 Fig. Individual variation in the linear Cesàro model. The optimized coefficients A and B in the linear Cesàro equation κ(s)=As+B, are presented in a 2D scatter plot. Plotting conventions are identical to S7 Fig.
